# Epoxy-Based Ion-Exchange Materials for Water Treatment and Resource Recovery: A Critical Review of Structure–Performance Relationships and Practical Limitations

**DOI:** 10.3390/polym18212710

**Published:** 2026-11-01

**Authors:** Ainash Baidullayeva, Nesipkhan Bektenov, Kanat Sadykov, Nurbatyr Mykhametgazy, Gulzhan Abdraliyeva, Katira Serikbayeva

**Affiliations:** 1 Department of Engineering Disciplines and Good Practices, School of Pharmacy, Asfendiyarov Kazakh National Medical University, Almaty 050044, Kazakhstan; 2 Department of Chemistry, Abai Kazakh National Pedagogical University, Almaty 050010, Kazakhstan; bektenbna@gmail.com (N.B.);; 3 Laboratory of Ion Exchange Resins and Membranes, Institute of Chemical Sciences Named After A.B. Bekturov, Almaty 050010, Kazakhstan; 4 Department of Chemistry, Chemical Technology and Ecology, Faculty of Biotechnology and Chemical Technologies, Almaty Technological University, Furkat Street 348/4, Almaty 050061, Kazakhstan

**Keywords:** epoxy-based ion-exchange materials, glycidyl methacrylate, accessible adsorption capacity, structure–performance relationships, binding mechanisms, competitive adsorption, water treatment, regeneration, resource recovery

## Abstract

Epoxy- and glycidyl methacrylate (GMA)-based polymers offer versatile platforms for ion exchange, contaminant removal, and resource recovery. However, their reported adsorption capacities do not always reflect the number of functional groups that remain accessible and effective under practical treatment conditions. This review critically evaluates the relationships among polymer architecture, functionalization chemistry, binding mechanisms, mass transfer, and operational performance. Particular attention is given to the distinction between theoretical, total, accessible, and operational capacities. The literature indicates that increasing functional-group density does not necessarily improve adsorption performance because crosslinking, limited swelling, pore accessibility, and steric hindrance can restrict ion transport and site utilization. Mechanistic interpretations also remain uncertain in many studies, as ion exchange, coordination, and chemisorption are frequently inferred from equilibrium or kinetic model fitting without sufficient counter-ion balance or direct spectroscopic evidence. Furthermore, adsorption performance is predominantly evaluated in single-solute laboratory systems, while systematic comparisons under competitive-ion conditions, authentic water matrices, and continuous-flow operation remain limited. Regeneration studies often demonstrate short-term capacity retention but provide insufficient information on ligand leaching, chemical consumption, recovered-product purity, and long-term durability. Direct benchmarking against commercial ion-exchange resins is also uncommon. This review therefore identifies accessible binding-site density, experimentally verified mechanisms, dynamic separation performance, and regeneration stability as essential criteria for evaluating epoxy-based ion-exchange materials. Future research should prioritize standardized testing, realistic feedwaters, extended regeneration cycles, and process-level comparisons to establish their practical feasibility and sustainability.

## 1. Introduction

Water contamination has become a major environmental, technological, and public-health challenge. Municipal wastewater, industrial effluents, mining drainage, and seawater contain diverse contaminants, including toxic metals, oxyanions, pharmaceuticals, and persistent organic compounds [1,2,3,4,5]. These pollutants can threaten aquatic ecosystems and human health, even at low concentrations. However, some dissolved species also represent valuable secondary resources. Precious metals such as palladium and gold, as well as uranium, can be recovered from industrial wastewater and other aqueous sources [6,7,8]. Water treatment should therefore extend beyond contaminant removal to include selective capture and resource recovery. This approach supports circular-economy principles and more sustainable resource management.

Conventional water treatment methods include chemical precipitation, coagulation, solvent extraction, membrane separation, electrochemical treatment, and adsorption [9,10]. Although these methods are widely applied, their effectiveness depends on contaminant concentration, water composition, and operating conditions. Precipitation may generate substantial sludge, while solvent extraction can require organic solvents and additional separation steps [9]. Membrane technologies offer efficient separation but may face limitations related to fouling, energy consumption, and selectivity [10,11]. Ion-exchange and chelating materials provide an alternative through selective interactions between dissolved species and functional groups immobilized on solid supports [12,13,14]. Functional polymeric sorbents are particularly attractive because their chemical composition, surface functionality, and porous structure can be tailored to specific separation requirements [12,13,14,15,16]. They can be fabricated as microspheres, porous monoliths, membranes, or magnetic composites. Nevertheless, high adsorption capacity in single-solute experiments does not necessarily ensure effective performance in complex water matrices. Competitive ions, organic matter, and operating conditions can substantially influence adsorption behavior and separation efficiency [13,14,15,16].

Epoxy-containing polymers provide a versatile platform for constructing ion-exchange and chelating materials. Their epoxide groups can react with amines, thiols, carboxylates, phosphonates, and other nucleophiles under relatively mild conditions [17,18]. Glycidyl methacrylate is especially useful because its polymerizable methacrylate group enables matrix formation, while the pendant epoxide group remains available for subsequent modification [19]. This separation between matrix formation and functionalization permits control over both physical architecture and chemical affinity [20]. Nevertheless, several questions remain unresolved. High functional-group density is often assumed to produce high capacity, although internal sites may be inaccessible because of limited swelling, steric hindrance, or diffusion resistance [21]. Pseudo-second-order kinetics are also frequently interpreted as proof of chemisorption, despite the limited mechanistic information provided by empirical kinetic fitting [22]. Similarly, Langmuir behavior is often presented as evidence of homogeneous monolayer binding, although heterogeneous polymer networks may fit several equilibrium models equally well [23]. Reported selectivity may also reflect differences in aqueous speciation, pH, or initial concentration rather than intrinsic ligand–ion affinity [24]. These uncertainties require chemical, structural, equilibrium, kinetic, and competitive evidence to be evaluated together. Recent studies have further demonstrated the potential of functionalized polymeric adsorbents, including GMA-based composites, for the removal of diverse aqueous contaminants [25,26]. Fixed-bed adsorption systems and adsorbent regeneration strategies are also important considerations for translating laboratory findings into practical water treatment applications [27,28].

This review provides a critical assessment of epoxy- and GMA-based ion-exchange materials beyond conventional classifications of polymer composition, functionalization methods, and adsorption applications. Its principal contributions are fourfold. First, it distinguishes theoretical, total, accessible, and operational capacities to explain why functional-group loading does not necessarily translate into effective ion uptake. Second, it critically examines the experimental evidence supporting ion-exchange, electrostatic, coordination, and chelation mechanisms, highlighting the limitations of equilibrium and kinetic model fitting. Third, it evaluates the effects of competing ions, complex water matrices, and dynamic operating conditions on the transferability of laboratory adsorption results. Fourth, it connects regeneration efficiency, material durability, resource recovery, and scale-up requirements to practical process performance. By integrating these perspectives, the review identifies the characterization and benchmarking approaches needed to establish reliable structure–performance relationships and assess the feasibility of epoxy-based materials for sustainable water treatment.

## 2. Materials and Methods

This study was conducted as a structured narrative review of epoxy-based ion-exchange and adsorption materials for water treatment and resource recovery. The scope encompasses epoxy-derived and glycidyl methacrylate (GMA)-based polymer systems, including functionalized epoxy resins, GMA-containing copolymers, grafted polymers, and hybrid composites in which epoxy/GMA chemistry contributes to material synthesis or functionalization. Materials without epoxy/GMA-derived components were considered only as comparative examples to illustrate relevant binding mechanisms or material-design principles and were not included in the primary performance comparisons. Literature searches were performed in Scopus, PubMed, Europe PMC, Google Scholar, and Embase between September and October 2026. The searches covered publications from 2015 to 2026 and combined terms related to epoxy resins, glycidyl methacrylate (GMA), poly(glycidyl methacrylate) (PGMA), and epoxide-functionalized polymers with terms related to ion exchange, adsorption, chelation, water purification, and resource recovery. Search strategies were adapted to the syntax and indexing functions of each database. Complete search strings, database-specific filters, retrieval counts, search dates, responsible researchers, and search links were documented (Table S1).

**Figure 1:**
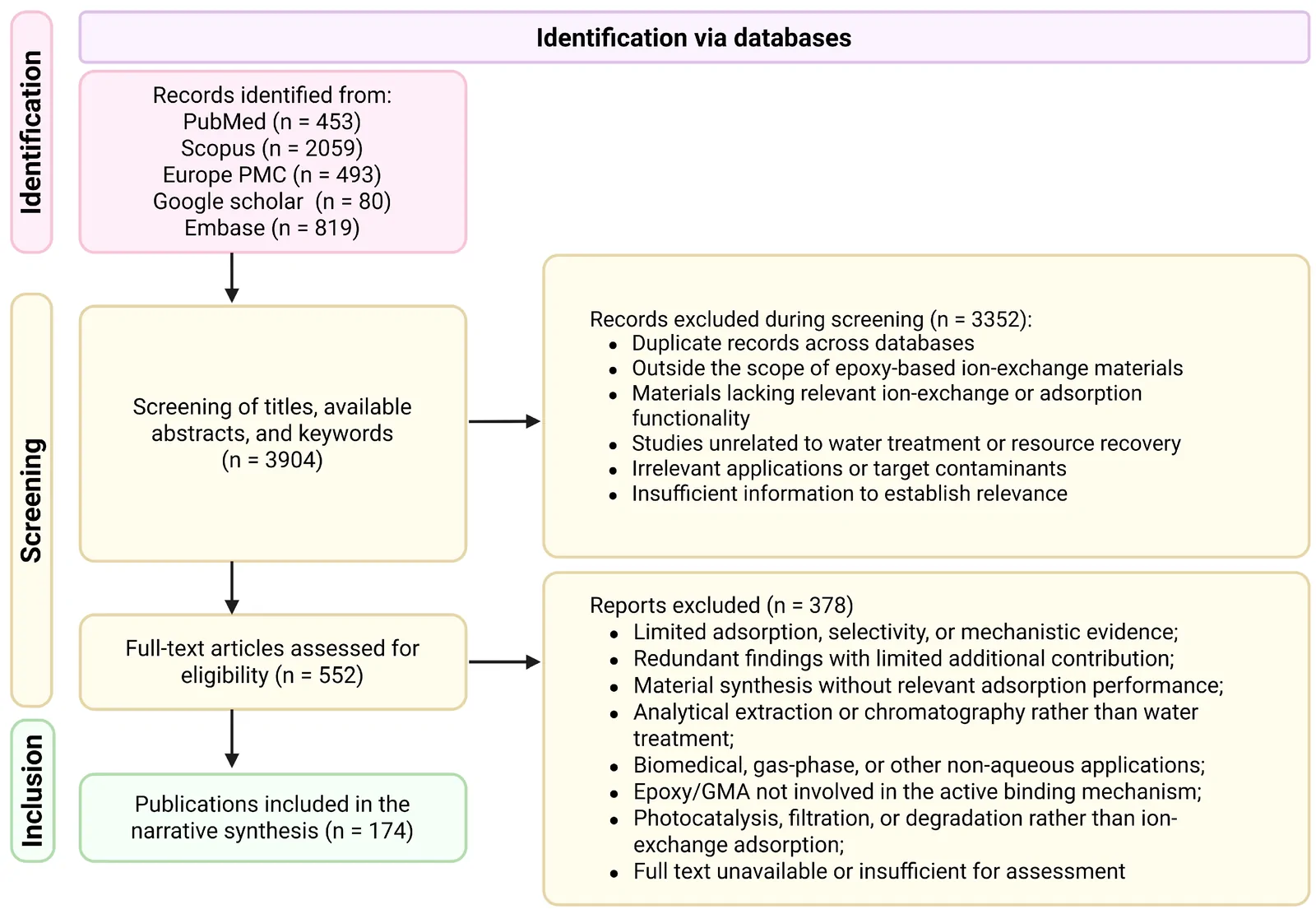
Classification of epoxy-based ion-exchange materials by polymer matrix and physical architecture. Created in BioRender. Ainash, B. (2026) https://BioRender.com/ewbarsh.

The selection process involved title, abstract, and keyword screening, followed by full-text eligibility assessment. Studies were considered relevant when they investigated epoxy-based or GMA-containing polymer systems for aqueous adsorption, ion separation, contaminant removal, or resource recovery. This included hybrid materials based on non-epoxy supports, provided that epoxy/GMA-derived chemistry formed an identifiable part of the functional material. Studies involving materials without epoxy/GMA-derived components were excluded from the core evidence synthesis but could be cited separately as contextual or comparative examples when necessary. Priority was given to studies reporting material composition, functionalization methods, adsorption performance, binding mechanisms, selectivity, or regeneration. Studies were excluded when the polymer chemistry or application fell outside the review scope, relevant adsorption data were insufficient, or the findings did not provide a meaningful contribution to the synthesis. Duplicate records and publications with overlapping data were also considered during screening. A total of 3904 records were identified, of which 3352 were excluded during screening. Subsequently, 552 reports underwent full-text eligibility assessment, 378 were excluded, and 174 publications were included in the narrative synthesis (Figure 1).

Relevant information was extracted and organized according to the main themes of the review. These included polymer architecture and functional groups, synthesis and characterization, adsorption and ion-exchange mechanisms, water treatment applications, and regeneration or resource recovery performance. Where available, the extracted data included experimental conditions, adsorption capacities, ion-exchange capacities, kinetic and equilibrium parameters, competitive-ion effects, and reuse performance. Studies were assessed for their relevance to individual manuscript sections, and inclusion or exclusion decisions were documented with supporting reasons (Table S2). Review articles were used primarily for background and contextual interpretation, while quantitative performance comparisons relied mainly on original research.

**Figure 2:**
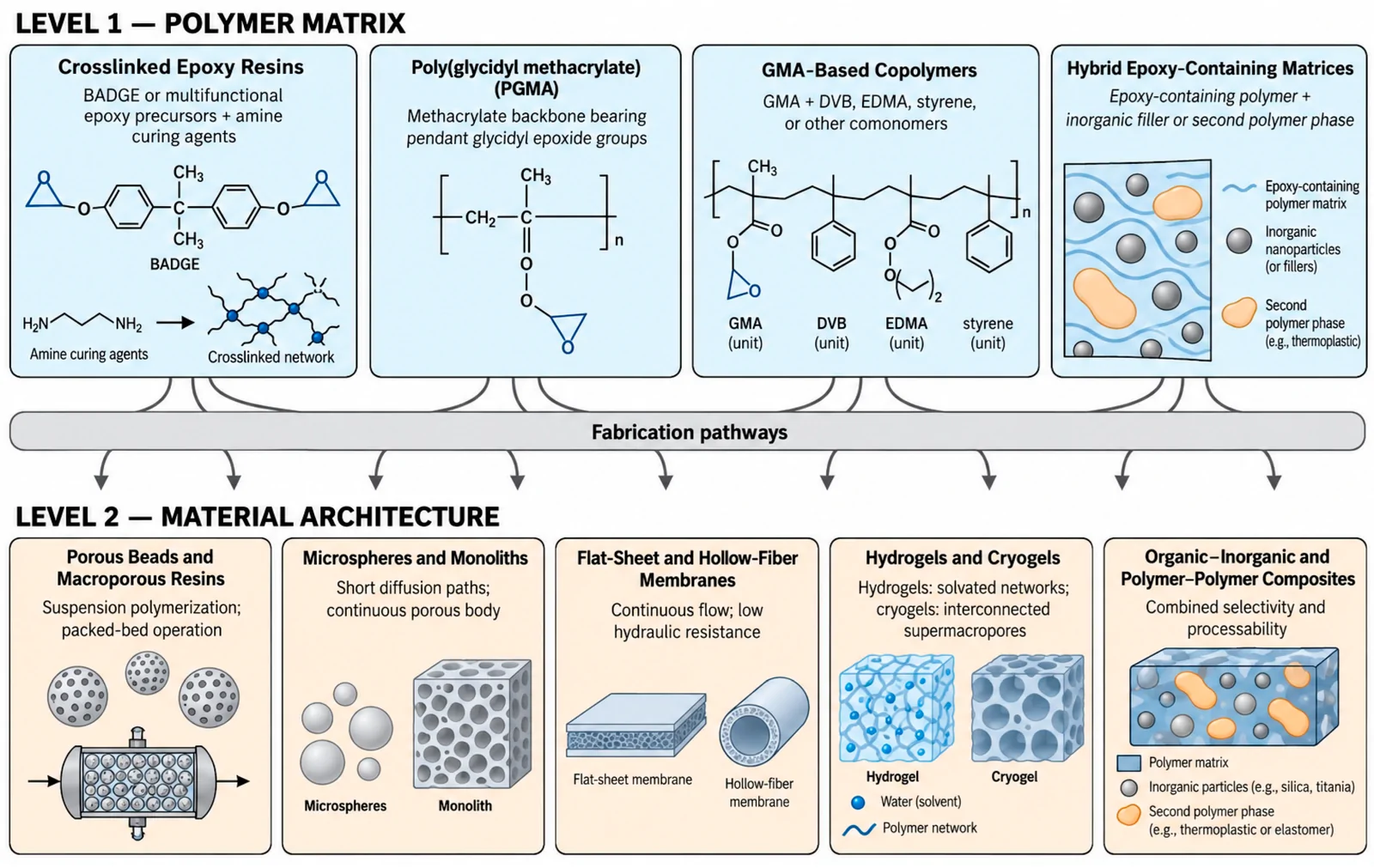
Classification of epoxy-based ion-exchange materials by polymer matrix and physical architecture. Level 1 (light blue) shows the principal polymer matrices, while Level 2 (light beige) presents their possible material architectures. The gray band and arrows indicate nonexclusive fabrication pathways between the two levels. Blue outlines mark epoxide groups, blue lines and circles represent hydrated polymer networks and water, gray spheres indicate inorganic fillers, and orange domains represent secondary polymer phases. All structures, pores, and particles are schematic and not drawn to scale.

**Figure 3:**
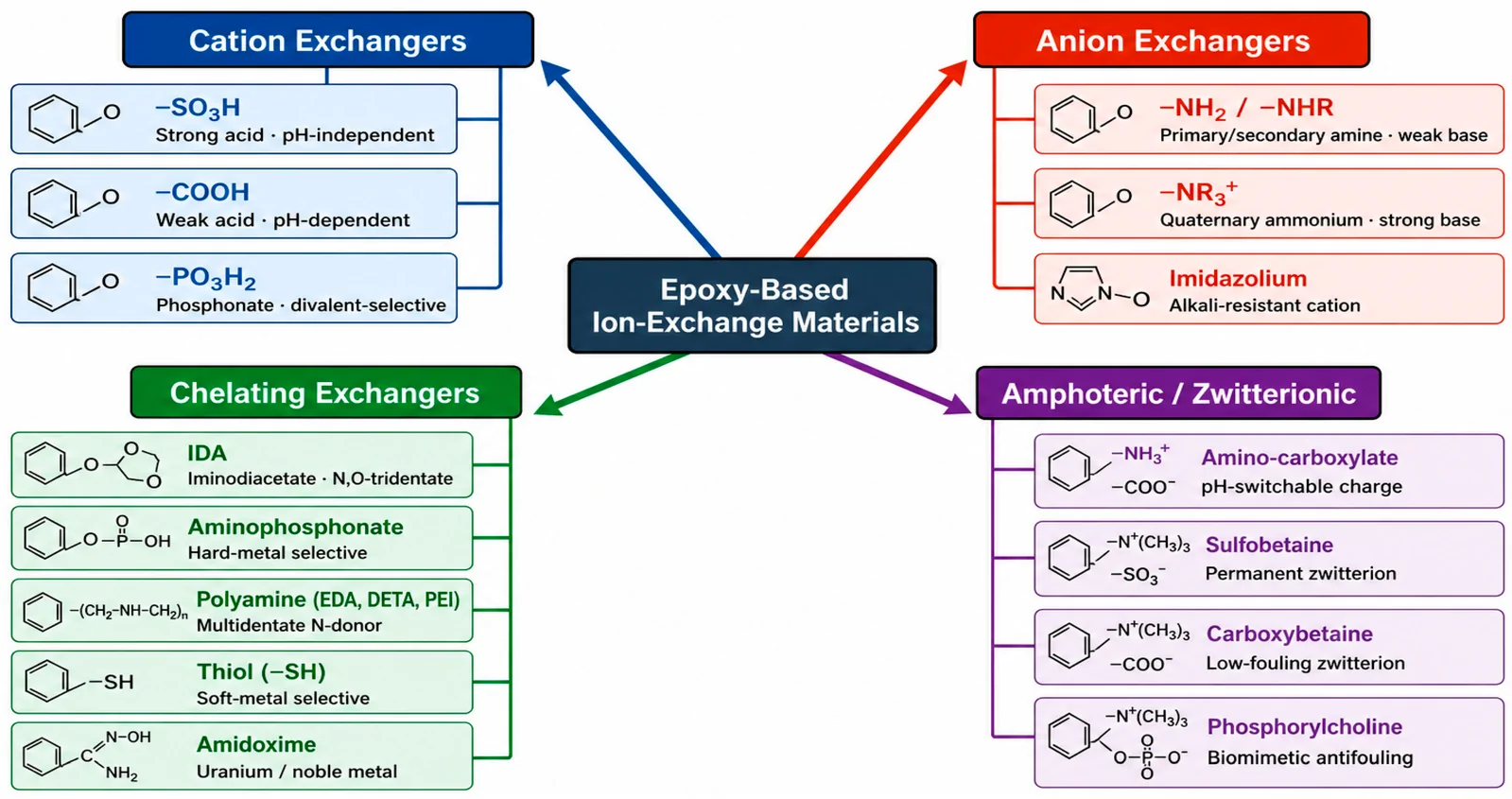
Classification and representative functional groups of epoxy-based ion-exchange materials. The materials are classified as cation exchangers containing sulfonic, carboxylic, or phosphonic acid groups; anion exchangers bearing amine, quaternary ammonium, or imidazolium groups; chelating exchangers incorporating multidentate ligands such as iminodiacetate, aminophosphonate, polyamine, thiol, or amidoxime groups; and amphoteric or zwitterionic exchangers containing oppositely charged functionalities. The chemical nature of these groups governs charge behavior, pH responsiveness, ion selectivity, and regeneration requirements.

**Figure 4:**
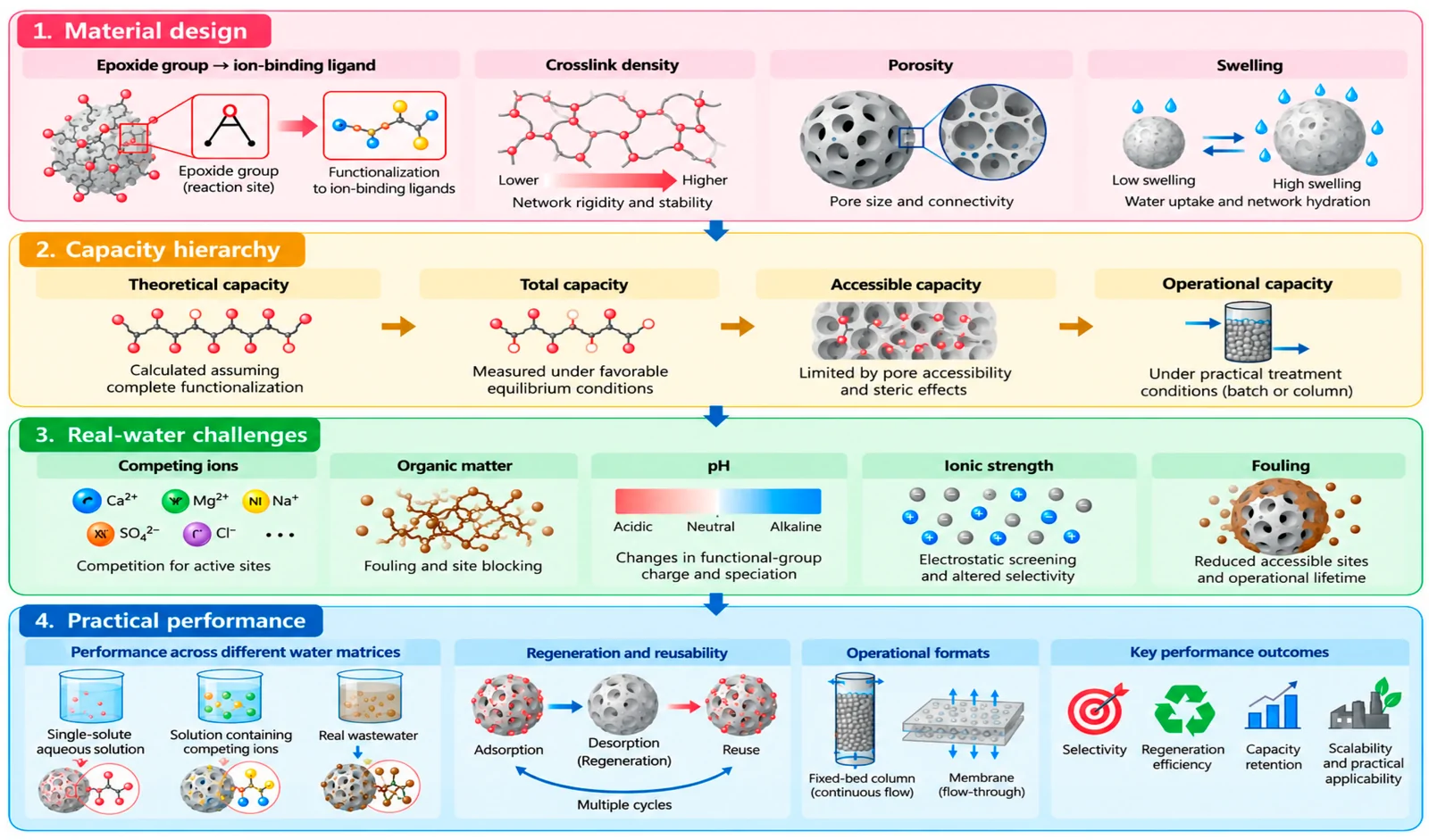
Conceptual framework linking material design, adsorption capacity, water-matrix effects, and practical performance of epoxy-based ion-exchange materials.

**Figure 5:**
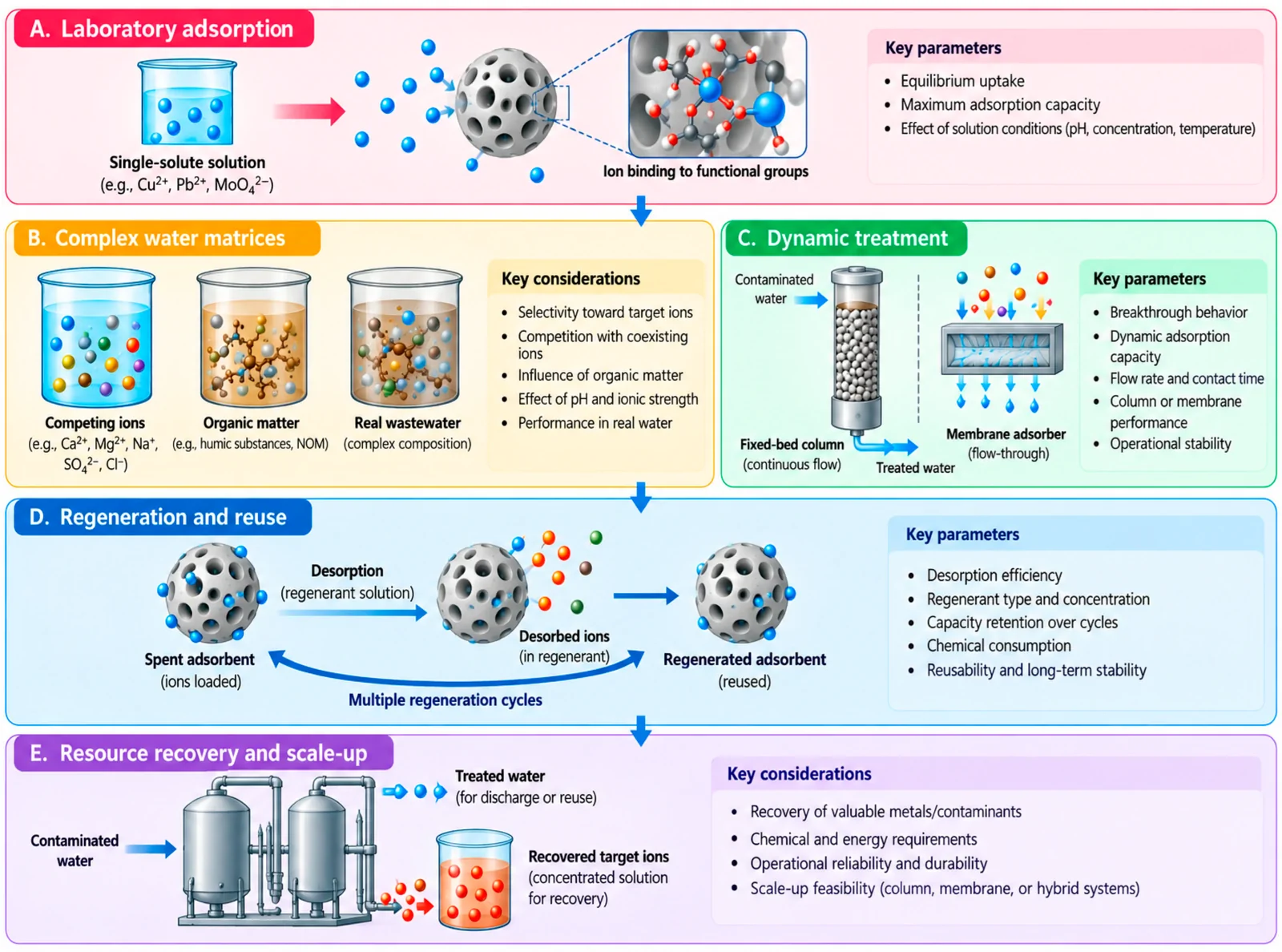
From Laboratory Adsorption to Practical Water Treatment, Regeneration, and Resource Recovery.

Evidence quality was evaluated qualitatively by considering the completeness of material characterization, clarity of experimental conditions, appropriateness of controls, and strength of the reported mechanistic evidence. Particular attention was given to whether proposed binding mechanisms were supported by spectroscopy, elemental analysis, ion-release measurements, or competitive adsorption experiments. Adsorption isotherm and kinetic model fits alone were not treated as definitive mechanistic evidence. Experimental uptake values were distinguished from model-derived maximum capacities, and missing data were not estimated. Regeneration, selectivity, and practical applicability were considered when evaluating material performance. Because the studies differed substantially in polymer composition, target contaminants, experimental conditions, and reported outcomes, the findings were synthesized narratively rather than through statistical meta-analysis. Figure 1 was prepared using BioRender (Science Suite Inc., Toronto, ON, Canada; https://www.biorender.com). Figure 2, Figure 3, Figure 4 and Figure 5 were developed with the assistance of ChatGPT (GPT-6; OpenAI, San Francisco, CA, USA; https://chatgpt.com) and its image-generation functionality for conceptual visualization, graphical design, and figure refinement. All figures, including their scientific content, labels, and graphical elements, were reviewed, verified, and edited by the authors, who take full responsibility for their accuracy and integrity.

## 3. Classification and Molecular Design of Epoxy-Based Ion-Exchange Materials

### 3.1. Epoxy-Based Polymer Matrices and Material Architectures

Epoxy-based ion-exchange materials form a structurally diverse family unified by reactive epoxide groups. These groups provide a versatile platform for introducing ion-binding sites and constructing three-dimensional polymer networks. The materials can be divided into four principal matrix classes. The first class consists of thermoset epoxy resins produced by curing multifunctional epoxy precursors, most commonly bisphenol A diglycidyl ether (BADGE) and related oligomers, with amines or anhydrides. The second class includes poly(glycidyl methacrylate) (PGMA) homopolymers. The third comprises random or block copolymers of GMA with comonomers that control crosslinking density, hydrophilicity, or structural rigidity [29,30]. The fourth class includes hybrid and composite matrices in which epoxide groups form reactive interfaces between a polymer and an inorganic or secondary polymer phase. All four classes exploit the strained oxirane ring. Its electrophilic carbon readily undergoes nucleophilic ring-opening by amines, thiols, phosphonates, and carboxylates. This reaction enables the direct introduction of ion-exchange and chelating groups after polymer synthesis [31,32]. Importantly, matrix chemistry is distinct from physical architecture. The same epoxy or GMA matrix can be fabricated as macroporous beads, microspheres, monoliths, membranes, hydrogels, cryogels, or composites. Each architecture produces different mass-transfer behavior and application suitability (Figure 2).

Thermoset epoxy networks are produced by curing multifunctional epoxy precursors with di- or polyamines. Their highly crosslinked three-dimensional structure provides resistance to acidic and alkaline regeneration media. It also improves hydrolytic stability and dimensional stability under changing osmotic conditions. These properties are important for repeated sorption and regeneration cycles in aqueous systems. Bicak et al. demonstrated that crosslinked epoxy networks prepared from conventional epoxy precursors and amine reagents could remove transition-metal cations together with their counteranions [29]. The observed bifunctionality was attributed to complementary interactions involving tertiary amine and residual hydroxyl groups. Similarly, Kornilov et al. prepared a chelating sorbent by reacting *o*-diaminobenzene with an epoxy resin [31]. The resulting material removed heavy metal ions and microplastics, indicating that the selected amine curative strongly influences the available interaction sites. Golubenko et al. extended thermally cured epoxy chemistry to anion-exchange membranes with defined mechanical and ion-transport properties [30]. However, extensive crosslinking can bury functional groups within the network and restrict their accessibility. Dense networks also increase diffusion resistance, reduce effective exchange capacity relative to the theoretical value, and may cause brittleness in particulate materials [29]. These limitations have encouraged the development of GMA-based matrices and more open physical architectures.

PGMA and GMA-based copolymers offer a versatile design strategy because polymer synthesis and functionalization can be performed in separate steps. During free-radical polymerization, the methacrylate double bond reacts, while pendant epoxide groups can remain available for subsequent modification [32,33]. This approach allows the polymer matrix to be formed before introducing ion-binding groups. Crosslinking density, porosity, swelling, and mechanical properties can be adjusted through the selection of comonomers and crosslinkers. GMA is commonly copolymerized with divinylbenzene (DVB) or ethylene dimethacrylate (EDMA). Other comonomers, such as styrene and hydroxyethyl methacrylate, can modify hydrophobicity and swelling behavior. The resulting matrices support different ion-binding mechanisms. Ybraimzhanova et al. synthesized phosphorus-containing ion exchangers from GMA, methyl methacrylate (MMA), and oxyethylidene diphosphonic acid using microwave-assisted processing. These materials achieved adsorption capacities of 40.3 mg/g for Cr(VI) and 40.4 mg/g for Pb(II) [32]. Li et al. quaternized poly(GMA-DVB) to produce a strong anion exchanger [33]. Wei et al. prepared sulfonated PGMA-DVB microspheres with cation-exchange functionality [34]. Li et al. developed poly(GMA-glucamine) through radical polymerization followed by functionalization with N-methyl-D-glucamine. The resulting material contained vicinal diol groups that enabled selective boron adsorption from seawater [35]. Shen et al. incorporated Fe_3_O_4_ nanoparticles into a PGMA-DVB matrix using seed swelling polymerization. Subsequent sulfonation introduced ion-binding groups and enabled selective La(III) adsorption [36]. These studies demonstrate how GMA-based matrices can accommodate different functional groups and target ions. However, adsorption performance depends on both ligand chemistry and the accessibility of binding sites. Excessive crosslinking or unfavorable functionalization conditions may restrict access to these sites and reduce effective adsorption capacity.

Physical architecture also influences adsorption performance by controlling surface accessibility, diffusion distance, hydraulic resistance, and mechanical stability. Porous beads and macroporous resins are commonly prepared through suspension polymerization using porogens. Their interconnected pores facilitate access to internal binding sites, while crosslinking provides structural stability. However, intraparticle diffusion can limit adsorption rates, particularly in larger particles [29,33]. Microspheres offer shorter diffusion paths and greater external surface area. Wei et al. demonstrated this approach using sulfonated PGMA-DVB microspheres [34]. Shen et al. further developed magnetic PGMA-DVB microspheres containing Fe_3_O_4_ nanoparticles and sulfonic acid groups. The particles showed selective La(III) adsorption, with a maximum capacity of 51.3 mg/g. They also retained 85% adsorption efficiency after five adsorption–desorption cycles [36]. Magnetic responsiveness facilitated particle recovery without conventional filtration. Nevertheless, small microspheres may increase pressure drop when used in packed-bed columns. Monoliths provide an alternative architecture with interconnected flow-through macropores. These channels promote convective transport and can reduce mass-transfer limitations compared with diffusion-dominated porous particles [37]. Therefore, selecting an appropriate architecture requires balancing functional-group accessibility, adsorption kinetics, mechanical stability, and practical separation requirements.

Membrane and gel architectures provide additional control over mass transfer. Flat-sheet and hollow-fiber membranes enable continuous-flow ion separation with relatively low hydraulic resistance. Golubenko et al. showed that crosslinked epoxy networks retain their structural integrity in flat-sheet anion-exchange membranes [30]. Composite membranes containing ion-exchange particles have also been applied to heavy metal removal and electrodialytic desalination [38]. Hollow fibers further increase the available membrane area per unit module volume [39]. In contrast, hydrogels use lightly crosslinked, highly solvated networks to maximize the accessibility of functional groups [40]. Cryogels contain interconnected, ice-templated supermacropores that may exceed 100 µm. These pores permit pressurized flow-through operation with minimal back-pressure. Türkmen et al. and Baimenov et al. demonstrated the advantages of cryogel architectures for heavy metal removal [41,42]. Malakhova et al. extended this approach to composite supermacroporous materials [37]. Despite their high accessibility and favorable transport properties, hydrogels and cryogels often have limited mechanical strength.

Organic–inorganic and polymer–polymer composites combine the functions of two or more phases. Inorganic ion-exchange particles, such as zirconium phosphate or cerium phosphate, can be dispersed within an epoxy-containing polymer matrix. Alternatively, a secondary polymer phase can be introduced to modify swelling, mechanical behavior, or selectivity. These combinations couple the selective binding properties of the dispersed phase with the processability of the polymer matrix [38,43,44,45]. Composite and hybrid membranes further adapt these advantages to continuous-flow separation systems [44,46]. Therefore, the selection of a matrix and architecture must consider the target ion, water composition, flow conditions, regeneration chemistry, and intended recovery process [29,30].

Several limitations remain across all material classes. First, terminology is inconsistent. Terms such as “macroporous resin,” “hydrogel,” and “monolith” are sometimes used interchangeably despite important morphological differences. This inconsistency complicates comparisons among studies. Second, direct comparisons of different architectures prepared from the same polymer matrix and carrying identical functional groups remain rare. Most studies change both matrix chemistry and architecture simultaneously. Consequently, the specific contribution of architecture cannot be isolated reliably [38,39]. Third, many materials are evaluated only in synthetic solutions containing a single target ion. Such conditions do not reproduce the competing ions, natural organic matter, and variable pH of real water matrices [44]. Finally, a persistent trade-off exists between accessibility and stability. Open, lightly crosslinked networks expose more exchange sites, whereas densely crosslinked networks provide greater mechanical durability. Resolving this trade-off will require standardized characterization methods and systematic comparisons of multiple architectures under equivalent operating conditions.

### 3.2. Classification by Ion-Exchange Functionality

The ion-exchange performance of epoxy-based materials depends primarily on the identity and density of their fixed functional groups. In epoxy and GMA matrices, the oxirane ring provides a reactive site for post-polymerization modification with heteroatom-containing nucleophiles. This approach allows charge type, charge density, and coordination environment to be adjusted without replacing the underlying polymer matrix. Based on their functional groups and interaction mechanisms, these materials can be classified as cation exchangers, anion exchangers, chelating exchangers, or amphoteric and zwitterionic exchangers. Cation exchangers contain fixed anionic groups that interact with dissolved cations. Anion exchangers contain fixed cationic groups that bind anions. Chelating materials use multiple nitrogen, oxygen, or sulfur donor atoms to coordinate metal ions. Amphoteric and zwitterionic materials contain both positive and negative sites within the same structure. The specific functional group determines the operating pH range, ion affinity, exchange kinetics, and regeneration requirements of each material (Figure 3).

Cation-exchange functionality is commonly introduced through sulfonate, carboxylate, or phosphonate groups. Sulfonate groups can be formed by opening GMA epoxide rings with bisulfite reagents or by directly sulfonating aromatic comonomers. The resulting strong-acid exchangers remain ionized across a broad pH range. El-Aassar et al. prepared sulfonated PGMA composites using azo dye-bearing sulfonated ligands. These materials removed methylene blue through cation-exchange interactions and were also applied to Cr(VI) and Mn(VII) removal [47,48]. A crystal-violet-immobilized SPGMA derivative was subsequently developed for treating chromate-containing industrial effluents under different operating conditions [49]. Sulfonation chemistry has also been applied to membrane architectures. UV-initiated grafting of GMA onto polyethersulfone substrates, followed by ring-opening sulfonation, produced adsorber membranes with competitive Cu(II) capacities at low operating pressures [50]. In another approach, sulfonated PGMA-maleic anhydride copolymers were grafted onto Fe_3_O_4_ nanoparticles. These composites combined Cu(II) uptake with magnetic separation and could be regenerated using dilute acid [51]. Weak-acid exchangers can be obtained by introducing carboxylic acid groups through hydrolysis or copolymerization with methacrylic acid. Their capacity is strongly pH-dependent because the carboxyl groups must be deprotonated to bind cations effectively [52]. Phosphonate-functionalized GMA materials provide another option for divalent and multivalent cation binding. However, this group remains less extensively investigated than sulfonate and carboxylate functionalities.

Anion-exchange sites are introduced mainly by reacting epoxide groups with primary or secondary amines. Subsequent N-methylation or N-alkylation can convert tertiary amines into permanently charged quaternary ammonium groups. Imidazolium groups provide another possible source of fixed positive charge. Du et al. grafted GMA onto cotton linter by radiation-induced polymerization. Ring-opening with pentaethylenehexamine and subsequent methylation produced a quaternary ammonium material with a phosphate capacity exceeding 80 mg P g^−1^ under dynamic column conditions [53]. Tang et al. prepared magnetic methyl methacrylate and GMA copolymer microspheres containing two types of quaternary ammonium sites. The material simultaneously removed nitrate-N and phosphate-P from secondary municipal effluent [54]. Amine-functionalized macroporous GMA microspheres have also been used for fluoride removal from aqueous solutions [55]. Their protonatable primary amine groups support anion binding under mildly acidic conditions. Feng et al. used diglycidyl ether spacers to connect quaternary ammonium groups to PS-DVB/Fe_3_O_4_ composite beads. The resulting branched networks showed rapid nitrate adsorption and could be recovered magnetically after treatment [56]. Overall, quaternary ammonium groups provide strong anion-exchange capacity across a relatively broad pH range. In contrast, primary, secondary, and tertiary amines show pH-dependent charge and selectivity.

Chelating exchangers use coordinated metal-ligand interactions to achieve greater selectivity than conventional electrostatic exchange. Iminodiacetate (IDA) is one of the most extensively studied chelating groups on GMA-based supports. It contains two carboxylate groups and one amine nitrogen that can interact cooperatively with metal ions. An et al. reported Cu, Pb, and Cd capacities of 1.2 to 2.5 mmol g^−1^ for an IDA-modified D301 resin [57]. The observed affinity pattern was associated with combined carboxylate attraction and nitrogen coordination. Jun et al. prepared GMA-IDA resins and evaluated their binding of Cd, Cu, Ni, Zn, and Pb [58]. Their results showed higher capacities than those of simple carboxylate analogues, highlighting the contribution of the amine donor to complex stability. Fadel et al. and El-Bahy et al. also examined IDA resins for Cu, Ni, and Hg removal. These materials could be regenerated by acid elution while retaining their exchange capacity over repeated cycles [59,60].

Polyamine groups further expand the coordination chemistry of GMA-based exchangers. Common modifiers include ethylenediamine, diethylenetriamine, triethylenetetramine, tetraethylenepentamine, and polyethylenimine. Their multiple nitrogen atoms provide several potential coordination sites for transition and heavy metal ions. Gupta et al. functionalized GMA-styrene-MBA terpolymers with tetraethylenepentamine and reported an Hg(II) capacity of 2.8 mmol g^−1^ [61]. Suručić et al. compared poly(GMA-co-EGDMA) networks modified with ethylenediamine, diethylenetriamine, and triethylenetetramine [62]. Increasing the polyamine chain length improved Cu(II) capacity and broadened the effective pH range. GMA gel resins bearing tetraethylenepentamine groups also showed rapid Cu and Pb uptake because swelling shortened the diffusion pathways to the binding sites [63]. Mixed-ligand PGMA brushes provide additional control over selectivity. Surface-initiated atom transfer radical polymerization was used to prepare brushes containing 5-aminosalicylic acid and imidazole-derived groups. Their relative affinity for transition metals could be adjusted through pH control [64].

The recyclability of chelating materials depends on the stability of the immobilized ligands. Polyethylenimine-rich GMA copolymers maintained Cu and Pb removal over 15 regeneration cycles [65]. Ethylenediamine-functionalized PGMA brushes also retained Cu(II) and Cd(II) uptake over ten adsorption and desorption cycles [66]. Higher-denticity ligands can further increase capacity. Functionalization of a GMA-based polymer with diethylenetriamine tetraacetic acid produced a Cu(II) capacity of 3.1 mmol g^−1^ at low feed concentrations [67]. These findings demonstrate the advantages of multidentate coordination. However, high ligand density can increase synthesis complexity, steric crowding, and material cost.

Amphoteric and zwitterionic materials contain both positive and negative functional groups. Their charge state may change with pH, allowing the same material to interact with cationic and anionic species. Amphoteric GMA networks can be prepared by introducing both amine and carboxylic acid groups through sequential ring-opening and hydrolysis reactions. Below the isoelectric point, protonated amine groups promote anion exchange. Above the isoelectric point, deprotonated carboxyl groups favor cation exchange. Permanent zwitterionic groups, including sulfobetaines and carboxybetaines, retain both charges over a wider pH range. Ruan et al. grafted sulfobetaine methacrylate onto polydopamine-coated anion-exchange membranes. The resulting zwitterionic surfaces reduced organic fouling while preserving ion-exchange capacity during electrodialysis [68]. Composite membranes based on poly(GMA-co-mPEGMA) crosslinked with polyethylenimine combined electrostatic and chelating interactions. These membranes simultaneously rejected anionic dyes and adsorbed heavy metal cations from complex effluents [69]. GMA-functionalized galactoglucomannan hydrogels also removed Cu(II), Cr(VI), and As(V) through mixed interaction mechanisms [70]. In addition, lysine-modified PGMA membranes introduced amino and carboxylate groups through epoxide ring-opening. These materials were applied to selective lanthanide recovery from dilute aqueous solutions [71]. Together, these examples illustrate how the four functional classes differ in fixed charge, binding mechanism, and representative target ions (Figure 3).

Each functional class offers a distinct balance between selectivity, operating stability, and synthesis complexity. Strong-acid sulfonate exchangers provide rapid exchange and a broad operating pH range. However, they generally show limited ion specificity and may bind abundant Ca^2+^ and Mg^2+^ ions in real water. Weak-acid carboxylate exchangers can favor divalent ions over monovalent ions at suitable pH values. Their capacity decreases under acidic conditions because the carboxyl groups become protonated. Quaternary ammonium exchangers are effective for nitrate, phosphate, arsenate, fluoride, and other anions. Nevertheless, natural organic matter can foul these materials and reduce their accessible capacity. Chelating exchangers provide greater metal-ion selectivity through inner-sphere coordination. Their preparation often requires several functionalization and purification steps, which can increase cost and batch variability. Amphoteric and zwitterionic materials combine mixed-ion removal with potential antifouling properties. However, their performance has been evaluated mainly in laboratory-scale batch or membrane experiments. Future studies should compare these functional classes under standardized conditions and in realistic water matrices. Such comparisons are essential for separating the effects of functional-group chemistry, matrix structure, and material architecture.

### 3.3. Molecular and Structural Design Parameters

The performance of epoxy-based ion-exchange materials depends on several interconnected structural variables. These include pore architecture, pore-size distribution, crosslink density, functional-group density, and the spatial distribution of active sites. Their effects can be understood through four levels of capacity. Theoretical capacity is calculated from monomer stoichiometry and assumes complete functionalization. Total capacity is measured under favorable equilibrium conditions but may be reduced by incomplete conversion or burial of reactive sites. Accessible capacity includes only the functional groups that hydrated ions can reach. Operational capacity is further affected by flow conditions, fouling, and regeneration efficiency. In DVB-GMA porous polymers, changing the crosslinker-to-monomer ratio simultaneously alters surface area, pore volume, and epoxide concentration [72,73]. Therefore, these variables cannot be optimized independently. Effective material design requires a coordinated balance among chemical capacity, molecular accessibility, transport, and mechanical stability [74,75,76,77].

According to the IUPAC classification, pores are categorized as micropores below 2 nm, mesopores between 2 and 50 nm, and macropores above 50 nm. Each category contributes differently to ion accessibility and transport. DVB-GMA polymers have shown bimodal mesopore distributions at approximately 3.7 nm and 5.5 to 7.5 nm. Their BET surface areas ranged from approximately 140 to 190 m^2^ g^−1^ in less crosslinked formulations and from approximately 320 to 370 m^2^ g^−1^ in more highly crosslinked materials [72]. Porogen composition also controls the resulting pore network. Optimization of the toluene-to-3-methyl-1-butanol ratio produced a dynamic saturation capacity of 55.4 mg mL^−1^ of wet resin [77]. This performance was associated with improved pore connectivity rather than the highest absolute surface area. However, BET measurements based on nitrogen adsorption do not directly represent ion-accessible surface area in water. Hydrated divalent ions commonly have effective radii of approximately 0.4 to 0.8 nm. These ions may be excluded from narrow pore entrances even when nitrogen molecules can access the internal surface. Inorganic fillers can also block pores and reduce accessible pore volume [76]. Moreover, intraparticle diffusion remained rate-limiting in amino-functionalized GMA-EGDMA monoliths despite their high equilibrium capacity [75]. In contrast, a silica-supported cation exchanger with a moderate BET area of approximately 79.2 m^2^ g^−1^ achieved an exchange capacity of 1.41 meq g^−1^. It also exhibited approximately 12-fold faster kinetics than a commercial resin [74]. This result confirms that pore connectivity and framework stability can be more important than maximizing surface area alone.

Crosslink density regulates swelling, mechanical rigidity, and diffusion through the polymer network [78]. DVB and EGDMA are commonly used for this purpose. In polystyrene-DVB ion exchangers, increasing crosslinking reduces water uptake and hydrated-chain mobility [79,80]. This effect restricts counterion transport to functional groups located within the network. However, insufficient crosslinking can cause excessive swelling and poor dimensional stability. An intermediate crosslink density may therefore provide the best performance. For example, optimized DVB content in pore-filled ionomer membranes produced a desalting efficiency of 88.5%, compared with 78.7% for a commercial membrane [78]. Molecular dynamics simulations of DGEBA/MTHPA epoxy networks provide additional mechanistic support. Increasing crosslink density reduced mean-square displacement and fractional free volume while increasing glass-transition temperature and elastic modulus [81,82]. These changes improve rigidity but limit the segmental mobility required for ion diffusion. In methacrylate xerogels, approximately 1% EGDMA was required to form a coherent network. Further increases reduced equilibrium water content and increased shear modulus [83]. Crosslinking performance also depends on the chemistry and location of the crosslinks. In radiation-grafted anion-exchange membranes, diamine crosslinking during amination produced a better balance of ionic resistance and permselectivity than DVB crosslinking during graft polymerization [81]. Thus, crosslink density, crosslink chemistry, and spatial placement must be considered separately.

The degree of functionalization must be distinguished from accessible ion-exchange capacity [84,85,86]. The former describes the fraction of reactive epoxide groups converted into functional groups [84,87,88]. It can be assessed using FTIR, XPS, and quantitative chemical analysis. Accessible capacity, however, depends on whether the introduced groups remain available for ion binding under aqueous conditions. Spectroscopic evidence of functionalization does not necessarily establish their accessibility. Kanjilal et al. investigated amine functionalization of PGMA grafted onto polypropylene nonwoven fabric. After optimizing grafting and amination conditions, the DETA-functionalized material achieved a Pb(II) saturation capacity of approximately 230 mg g^−1^ [86]. Limsuwan et al. prepared IDA-functionalized PGMA grafted onto bacterial cellulose. The material exhibited an epoxide-to-IDA conversion of 37.47 ± 0.15% and an IDA group density of 1.52 ± 0.09 mmol g^−1^ [87]. In comparison, the corresponding polypropylene-based material showed a conversion of 20.61 ± 0.22% and an IDA density of 1.04 ± 0.02 mmol g^−1^. These findings demonstrate that polymer substrate properties influence functionalization efficiency. Other studies have identified additional structural constraints. In IDA-functionalized coatings, ligand density increased from 1.47 to 49.05 nmol cm^−2^ as epoxide content increased from 22 to 91 mol% [88]. The nonlinear relationship suggests that increasing epoxide content does not produce a proportional increase in ligand loading. Spacer length also affects binding-site accessibility. Ligands attached through spacer arms containing at least six atoms achieved static binding capacities above 150 mg mL^−1^, whereas shorter spacers yielded lower capacities [85]. GMA–EGDMA copolymers showed concurrent changes in surface area, pore volume, and epoxide content when synthesis conditions were varied [84]. Therefore, functional-group conversion, ligand density, and adsorption capacity should be evaluated separately. Combining spectroscopy with porosimetry, swelling measurements, and direct adsorption tests provides a more complete assessment of material performance.

The spatial distribution of functional groups provides another level of control. Active sites may be distributed throughout the polymer bulk, concentrated on the external surface, immobilized along pore walls, or arranged as brushes, gradients, and core–shell structures. Surface-localized functionalization shortens diffusion distances and can improve uptake kinetics. For example, AGET-ATRP was used to prepare epoxy-terminated brushes on regenerated cellulose membranes. Internal pore grafting was suppressed by filling the pores with a high-viscosity solvent, which concentrated the reactive brushes near the external surface [89]. Gradient distributions can provide additional benefits. In porous anion-exchange resins, long-chain quaternary ammonium groups were placed near the exterior, while shorter-chain groups were distributed within the pores. This arrangement improved adsorption kinetics and bacterial capture relative to uniformly functionalized materials [90]. Hyperbranched quaternary amine resins for perrhenate and pertechnetate separation also benefited from cooperative surface and pore functionality [91]. In membrane adsorbers, distributing the functional polymer throughout the pore cross-section reduced diffusion distances. These materials exhibited sorption rates up to two orders of magnitude higher than bead-based or surface-only systems [92]. Controlled polyelectrolyte nanolayers also produced high dynamic binding capacities for large proteins. In these systems, graft-chain spacing governed convective accessibility [93]. Small-angle neutron scattering revealed further nanoscale organization in graft-type imidazolium membranes. Their ionic domains changed between reverse-micelle-like and micelle-like structures depending on hydration [94]. These changes influenced water uptake and ion transport. Therefore, bulk functional-group density alone cannot describe material performance.

Pore structure, crosslinking, functionalization, and site distribution produce several competing effects. Greater porosity generally improves mass transfer but may weaken the material. Higher crosslink density improves dimensional stability but restricts swelling and diffusion. Increasing functional-group loading raises theoretical capacity, yet steric crowding and electrostatic exclusion may reduce the accessible fraction. Surface-localized sites improve uptake rates but can limit gravimetric capacity because much of the internal matrix remains unused. These relationships explain why the material with the highest surface area or functional-group content does not necessarily provide the highest operational capacity.

Several research gaps limit rational optimization. First, standardized methods for measuring accessible capacity under realistic flow conditions are lacking. Second, pore-size categories have not been systematically correlated with hydrated-ion accessibility, breakthrough behavior, and intraparticle diffusivity within a common material platform. Third, optimal crosslinking ranges have not been established for sulfonate, quaternary ammonium, and iminodiacetate groups in GMA-DVB and GMA-EGDMA networks. Fourth, the effect of network confinement on epoxide ring-opening has not been evaluated using combined spectroscopy, titration, and activity-based assays. Fifth, gradient and core–shell distributions remain underexplored in particulate epoxy-based resins. Sixth, the effects of network rigidity on charge screening, ion-exclusion regions, and effective site valency remain poorly defined under realistic pH and ionic-strength conditions. Seventh, long-term mechanical and functional stability has not been adequately investigated in highly functionalized materials with low crosslink density. Finally, advanced techniques such as small-angle X-ray scattering, small-angle neutron scattering, positron annihilation lifetime spectroscopy, and cryogenic electron microscopy remain underused. Combining these techniques with standardized performance testing could clarify how nanoscale structure governs accessible and operational ion-exchange capacity.

## 4. Synthesis, Functionalization, and Characterization

### 4.1. Functionalization Strategies for Epoxy-Based Ion-Exchange Materials

The practical success of post-synthetic functionalization depends not only on the reactivity of the epoxide group but also on the reaction environment and the accessibility of the polymer-bound oxirane rings. Epoxide-ring opening can be conducted in aqueous, polar organic, or mixed solvent systems, with the choice of medium governed by the solubility of the nucleophile and the swelling behavior of the polymer. Amines generally react directly with epoxide groups, whereas less nucleophilic reagents may require catalysts, elevated temperatures, or adjusted pH. The nucleophile-to-epoxide ratio and reaction time must be sufficient to promote conversion without unnecessarily increasing reagent consumption or side reactions. This balance is particularly important in porous PGMA-based materials, where the modifying reagent must diffuse through the pore network before reaching internal epoxide groups [72,84]. Consequently, rapid surface conversion may coexist with incomplete functionalization in the particle interior. Water also has a dual role: it can swell hydrophilic matrices and facilitate reagent penetration, but it can simultaneously promote epoxide hydrolysis to vicinal diols, thereby consuming reactive groups without introducing the intended ligand.

A second design decision is whether the ion-binding ligand should be attached directly to the epoxy matrix or through an intervening spacer. Direct functionalization reduces the number of synthetic steps and can provide chemically stable covalent attachment. It is therefore attractive for comparatively small nucleophiles, including simple amines and thiols, that can readily reach the oxirane ring. However, direct attachment may position bulky ligands too close to a rigid polymer surface, where steric obstruction limits their accessibility and coordination geometry. Spacer-mediated functionalization addresses this limitation by separating the binding group from the matrix through a flexible molecular arm. In membrane adsorbers, spacer arms containing at least six atoms produced static binding capacities above 150 mg mL^−1^, whereas shorter spacers gave lower capacities, demonstrating that ligand presentation can be as important as total ligand loading [85]. Nevertheless, longer spacers may require additional coupling and purification steps and can increase chain mobility, nonspecific interactions, and production cost. Lin et al. used epoxide-functional photocurable coatings as reactive surfaces for covalent immobilization of iminodiacetic acid [88]. As the epoxide fraction increased from 22 to 91 mol%, the immobilized ligand density increased from 1.47 to 49.05 nmol cm^−2^, but the relationship was nonlinear, indicating that steric saturation limited proportional utilization of the additional reactive groups. These observations support the use of spacer-mediated attachment when a ligand requires conformational freedom, while direct attachment remains preferable when synthetic simplicity, low reagent consumption, and strong covalent retention are the primary requirements. Physical loading of a ligand may be simpler still, but it introduces a greater risk of leaching than covalent immobilization during repeated treatment and regeneration cycles.

Sequential functionalization provides a route to multifunctional materials without reproducing the single-function classifications discussed in Section 3.2. In this strategy, only a controlled fraction of the available epoxide groups is consumed during the first modification, leaving residual oxirane rings for a subsequent reaction with a chemically distinct ligand. Reaction order is critical because the first reagent can change polymer swelling, local charge, hydrophilicity, and the accessibility of the remaining epoxide groups. For example, initial introduction of an amine can create both an ionizable site and a secondary reactive center for further derivatization, whereas premature addition of a bulky chelating ligand may obstruct the penetration of the second reagent. GMA-based matrices have been functionalized with diethylenetriamine tetraacetic acid, polyamines, iminodiacetate-derived groups, and diol-containing ligands through single- or multistep routes [57,58,59,60,61,62,63,64,65,66,67]. Dual-function chelating resins similarly demonstrate that more than one coordination environment can be incorporated into a common polymeric support [64]. Where nucleophiles exhibit overlapping reactivity, protection–deprotection chemistry or orthogonal coupling reactions may be necessary to prevent uncontrolled cross-reaction and loss of functional specificity. However, every additional reaction introduces further washing, solvent use, yield loss, and batch variability. Residual epoxide content should therefore be measured after each stage rather than assumed from feed composition. Spectroscopic confirmation must also be combined with titration or another quantitative method because disappearance of an epoxide signal does not distinguish productive ligand attachment from hydrolysis or other nonfunctional side reactions [86,87,88]. Sequential modification is thus most defensible when the second functionality adds a clearly demonstrated capability that cannot be achieved through a simpler single-stage reaction.

The location at which functionalization is performed introduces an additional processing choice. Bulk modification exposes the entire porous material to a reactive solution and can generate high total ligand loading, but its success depends on reagent diffusion through the swollen network. Surface-confined modification shortens diffusion paths and simplifies contact between ions and active groups, although much of the internal matrix may remain chemically unused. Grafting approaches provide greater control over the location and density of functional chains. In a grafting-from strategy, polymer chains grow from surface-bound initiating sites, whereas grafting-to requires preformed chains to diffuse toward and react with the surface; the latter may therefore be limited by steric congestion at high grafting densities. Sengupta and Wickramasinghe used activator-generated electron-transfer atom-transfer radical polymerization to control the location of GMA grafting on regenerated-cellulose ultrafiltration membranes [89]. Filling internal pores with a high-viscosity medium suppressed grafting within the pore network and concentrated epoxy-functional brushes near the accessible external surface. Related membrane studies show that polymer nanolayer architecture and graft-chain placement can strongly affect the utilization of immobilized groups [92,93]. Conversely, functionalization along interconnected pore walls can combine a high internal loading with relatively short transport distances, provided that the reaction solution wets the pores uniformly. Such heterogeneous modifications require careful control of activation, mixing, washing, and removal of unreacted reagents. They also require reproducibility testing across different regions of a membrane or different batches of particles because a similar average elemental composition can conceal substantial spatial heterogeneity.

Process intensification may reduce the long reaction times and large solvent volumes associated with conventional batch functionalization. Among the strategies represented in the available literature, radiation-induced grafting, photoinitiated coating, controlled radical polymerization, and flow-through modification are particularly relevant. Radiation grafting enables reactive chains to be introduced onto preformed supports without conventional chemical initiators. Du et al., for example, used radiation-induced graft polymerization followed by amination and methylation to produce a quaternary-ammonium adsorbent for phosphate removal [53]. Photocurable copolymer coatings provide another rapid route for introducing surface epoxide groups before ligand immobilization [88]. Controlled radical methods such as surface-initiated ATRP and AGET-ATRP offer improved regulation of graft location and chain architecture [89]. Meanwhile, flow-through functionalization can promote convective delivery of reagents through porous membranes and monoliths, potentially decreasing concentration gradients that develop during static immersion [92]. Radiation-grafted membranes also demonstrate that the stage at which crosslinking and amination are performed can alter the resulting membrane structure: diamine crosslinking during amination and DVB crosslinking during graft polymerization do not produce equivalent networks [81]. Despite these advantages, process intensification should not be equated automatically with scalability. Radiation and controlled-radical techniques require specialized equipment or reagents, photochemical methods are constrained by light penetration, and flow-through modification requires uniform hydraulic distribution. Claims of improved processing should therefore be supported by reagent utilization, energy demand, processing time, and batch-scale data rather than reaction time alone.

Overall, no single functionalization route simultaneously maximizes conversion, selectivity, accessibility, environmental compatibility, and scalability. One-step modification reduces processing stages, solvent consumption, and purification requirements, but it offers limited control over multifunctionality and ligand distribution. Multistep approaches permit more sophisticated surface chemistry but increase the risks of incomplete conversion, side reactions, residual reagents, and cumulative material loss. Direct covalent attachment provides strong ligand retention, whereas spacer-mediated and grafted structures can improve accessibility at the cost of additional synthesis and characterization. Furthermore, several widely used modifiers, alkylating agents, catalysts, and organic solvents create toxicity and waste-management concerns that are rarely included in assessments of ion-exchange performance. A meaningful comparison should therefore report epoxide conversion, ligand incorporation efficiency, isolated material yield, solvent and water consumption, washing requirements, functional-group leaching, batch-to-batch reproducibility, and performance after regeneration. Aqueous functionalization and lower-temperature reactions may reduce environmental burdens, but only if they avoid excessive dilution, prolonged treatment, and wastewater generation. Likewise, highly controlled laboratory methods are valuable mechanistically but may be unsuitable for large-scale resin or membrane production if they depend on costly catalysts, oxygen-free conditions, or specialized irradiation equipment. Future development should prioritize robust reactions conducted in safer media, quantitative mass balances, recovery or reuse of unreacted reagents, and validation at scales beyond small laboratory batches. These criteria provide a more defensible basis for selecting a functionalization strategy than adsorption capacity alone and establish the connection between synthetic methodology and the characterization requirements discussed in the following section.

### 4.2. Characterization and Structure–Property Verification

Reliable characterization of epoxy-based ion-exchange materials requires more than demonstrating contact between a modifying reagent and the polymer. A defensible structure–property relationship must establish a sequential chain of evidence, beginning with consumption of reactive epoxide groups and incorporation of the intended ion-exchange or chelating functionalities, as demonstrated in modified PGMA-based resins, membranes, and grafted supports [95,96,97,98]. This chemical evidence must then be complemented by surface and bulk analyses capable of determining the chemical state, quantity, and spatial distribution of the incorporated groups [99,100,101,102]. Subsequently, microscopy, porosimetry, wettability measurements, and thermal analysis are required to determine whether functionalization preserves or alters the morphology, pore network, hydration behavior, and structural stability of the material [103,104,105,106,107,108]. Finally, static capacity measurements, fixed-bed breakthrough experiments, regeneration tests, and repeated-cycle studies are needed to establish whether the identified functional groups remain accessible and operational under aqueous treatment conditions [109,110,111,112,113,114,115,116]. Because no individual technique provides evidence across all these structural and functional levels, complementary characterization is essential. In particular, the presence of a functional group should not be equated with its accessibility, and pollutant-specific adsorption capacity should not automatically be interpreted as intrinsic ion-exchange capacity (Table 1).

Spectroscopic analysis provides the first level of verification by determining whether the chemical structure changed after functionalization. FTIR is widely used because epoxide-ring opening can be followed through attenuation or disappearance of characteristic oxirane-associated bands, accompanied by the appearance of signals assigned to newly introduced amine, sulfonate, carboxylate, or phosphonate groups. This approach supported the conversion of radiation-polymerized PGMA into an anion exchanger for radioiodide uptake [95] and the sulfonation of PGMA–DVB microspheres [34]. Likewise, FTIR changes were used to evaluate amine modification of GMA-containing microspheres and post-functionalization of DMN-co-GMA materials [114,115]. Combining FTIR with Raman spectroscopy provided stronger structural evidence for sulfonated poly(acrylonitrile-co-GMA)/PVC membranes, for which ion-exchange capacities of 0.18–0.48 meq g^−1^ were subsequently measured [97]. Solid-state ^13C NMR can provide more direct information about changes in the carbon environment and was combined with FTIR-ATR to support epoxide-ring opening in a DETA-functionalized GMA–EGDMA monolith [75]. Nevertheless, the interpretation of spectroscopic data requires caution. Reduced peak intensity may reflect incomplete conversion, overlapping bands, sampling heterogeneity, or hydrolysis rather than productive ligand attachment. In addition, FTIR and Raman generally provide qualitative or semiquantitative evidence and cannot determine whether the introduced groups are accessible to hydrated ions. Spectroscopy should therefore verify chemical transformation but should not serve as the sole evidence of successful ion-exchanger preparation.

Surface-sensitive and bulk-composition techniques provide the second level of evidence by determining where the new functionality is located and in which chemical state it exists. XPS can distinguish nitrogen, sulfur, phosphorus, oxygen, and metal environments through survey and high-resolution spectra, making it particularly useful for confirming surface amination, sulfonation, or metal–ligand coordination. Depth-profiling analysis of post-modified PGMA brushes demonstrated that small amine modifiers could penetrate throughout the brush layer, whereas bovine serum albumin remained concentrated near the surface [99]. This finding illustrates that reagent size can produce substantially different spatial distributions even when both modifiers react with the same polymer-bound epoxide groups. Similarly, XPS combined with ICP-OES was used to distinguish surface coordination from bulk uptake of Cu^2+^, Cd^2+^, and Pb^2+^ by functionalized poly(GMA-co-EDMA) [100]. Studies of amino-modified PGMA microspheres and uranium-binding PGMA materials further combined XPS with CHN analysis to relate nitrogen incorporation to metal coordination [101,102]. Complementary characterization of EDA-modified, GMA-grafted cotton using FTIR, XPS, EDX, SEM, and elemental analysis provided more convincing evidence than any individual method [98]. However, XPS generally probes only the outer nanometres of a material, while elemental analysis provides a bulk average without revealing whether groups are located on the external surface, pore walls, or inaccessible internal domains. EDX offers useful spatial mapping but has limited sensitivity for light elements and should not replace quantitative elemental analysis. Therefore, agreement between surface and bulk measurements is essential, particularly for porous particles and thick membranes in which functionalization may be diffusion-limited.

Quantitative verification must then distinguish residual epoxide content, total functional-group incorporation, intrinsic ion-exchange capacity, and pollutant-specific adsorption capacity. These values represent different properties and should not be used interchangeably. Titrimetric epoxide determination has been applied to poly(GMA-co-EGDMA) materials to quantify unreacted oxirane groups as a function of monomer composition, crosslinking, and porogen conditions [84]. After functionalization, acid–base, potentiometric, conductometric, or probe-ion exchange measurements can estimate the concentration of ionizable sites. For thermally cured epoxy-based anion-exchange membranes, reported IEC values ranged from 1.5 to 3.7 mmol g^−1^, while water uptake varied from 20.0% to 330%, demonstrating that chemical capacity and hydration changed substantially across the membrane series [30]. By comparison, sulfonated poly(acrylonitrile-co-GMA)/PVC membranes exhibited IEC values of 0.18–0.48 meq g^−1^ [97]. Pollutant-specific batch capacities provide a further, but less intrinsic, measure of accessible functionality. Sulfonated PGMA–DVB microspheres reached a reported Cu^2+^ capacity of 75.08 mg g^−1^ [34], while PGMA materials containing different chelating groups exhibited Nd^3+^ capacities of 0.304–0.736 mmol g^−1^ [96]. Amine-functionalized GMA resins showed maximum Th^4+^ capacities of 50 and 64 mg g^−1^ [116]. Such values are useful for comparative application assessment, but they also depend on pH, ionic strength, ligand speciation, competing ions, dose, equilibration time, and the selected isotherm model. Consequently, adsorption capacity cannot independently establish intrinsic IEC or active-site density. Quantitative characterization should report the probe ion, counterion form, dry- or wet-mass basis, equilibration conditions, and calculation method.

Morphological and porous-structure measurements provide the next link between chemical composition and accessible performance. SEM reveals particle shape, membrane cross-sections, macroscopic pores, defects, and surface damage, but it does not independently quantify molecular-scale pore-size distributions. For sulfonated PGMA–DVB microspheres, microscopy showed mean particle diameters of 2.18 µm for PGMA and 2.52 µm after DVB incorporation, together with increased surface roughness [34]. AFM and confocal microscopy can complement SEM by characterizing surface topography and the distribution of coatings or grafted layers. For example, multilayer dextran decoration of poly(GMA-co-DVB) matrices changed the reported water-contact angle from 153° to approximately 0°, confirming a pronounced alteration of the interfacial character [105]. Gas adsorption and mercury intrusion provide quantitative information about dry-state surface area, pore volume, and pore-size distribution. DVB–GMA polymers containing different monomer ratios exhibited BET areas ranging from approximately 140–190 to 320–370 m^2^ g^−1^, with pore modes near 3.7 and 5.5–7.5 nm [72]. A histidine-modified poly(GMA-co-EGDMA) material retained a BET surface area of approximately 90 m^2^ g^−1^ [103]. However, functionalization can narrow or block pores, and drying can distort the structure of swollen ion exchangers. Thermal drying of a macroreticular resin caused particle shrinkage and pore collapse, whereas freeze-drying preserved the porous structure more effectively [106]. Moreover, a post-crosslinked resin reached a dynamic saturation capacity of 55.4 mg mL^−1^ of wet resin even though dry-state measurements alone could not explain its operational behavior [77]. Thus, BET surface area should be treated as a structural descriptor rather than a direct measure of hydrated-ion accessibility.

Because ion exchange occurs in aqueous media, dry-state characterization must be complemented by measurements under hydrated conditions. Water uptake and swelling influence network expansion and ion transport, whereas excessive hydration may compromise dimensional and mechanical stability. Crosslinked epoxy anion-exchange membranes have shown water uptake ranging from 20.0% to 330%, highlighting the influence of polymer composition and crosslinking [30]. Contact-angle measurements assess surface wettability, while streaming potential and zeta potential provide information on pH-dependent interfacial charge. However, contact angles are influenced by surface roughness, chemical heterogeneity, and water absorption. Zeta potential reflects charge at the electrokinetic slipping plane rather than the total density of internal ion-exchange sites. Therefore, neither measurement alone establishes accessible ion-exchange capacity. Thermal and mechanical analyses provide complementary information on material stability. TGA and DSC characterize thermal degradation and transitions, whereas tensile, compression, and dynamic mechanical tests evaluate structural integrity. Sulfonated PGMA-grafted membranes and amine-functionalized GMA resins have been characterized using thermal analysis [108,115,116]. However, thermal stability does not necessarily predict resistance to repeated exposure to acids, bases, salts, oxidants, or natural organic matter. Hydrated dimensional stability, mechanical durability, and chemical-aging tests are therefore essential for assessing performance under water-treatment conditions.

Dynamic testing provides a stronger approximation of practical performance than equilibrium batch measurements because it integrates accessible capacity, fluid transport, diffusion, and material stability. Fixed-bed breakthrough curves determine treated volume, breakthrough time, dynamic capacity, mass-transfer-zone development, and sensitivity to flow rate. For poly-GMA–glucamine used for boron recovery from seawater reverse-osmosis concentrate, bed-depth service-time analysis produced reported bed capacities of 3872–3999 mg L^−1^ and critical bed depths of 4.7–9.2 mm [109]. Regeneration using 1.0 M HCl achieved 97.5% desorption, produced a 78-fold boron enrichment, and retained more than 95% capacity over five cycles [109]. Likewise, a radiation-grafted PP-g-GMA column reportedly treated more than 4000 bed volumes of a 3 mg L^−1^ Cr(VI) solution before breakthrough at 155 BV h^−1^ [110]. The same material retained more than 94% adsorption efficiency through five cycles, although different regenerants were required for Cr(VI) and Cd(II) [110]. Aminated poly-GMA beads were also evaluated for dynamic phosphate removal and NaCl regeneration, with sulfate identified as an important competing ion [111]. A low-pressure GMA-grafted PES membrane adsorber achieved a dynamic Cu^2+^ capacity of 64.05 ± 0.60 mg g^−1^ of grafted material at a flux of 135 L m^−2^ h^−1^ and an operating pressure below 0.15 bar [50]. These studies show why static capacity alone is insufficient: a material may exhibit high equilibrium uptake yet perform poorly under flow because of slow diffusion, pressure buildup, pore blockage, or insufficient residence time.

Ultimately, structure–property verification should be evaluated as a convergent body of evidence rather than a checklist of instruments. At minimum, a new epoxy-based ion exchanger should be supported by chemical verification of functionalization, quantitative determination of residual epoxide and functional-group content, surface and bulk compositional analysis, wet- and dry-state structural characterization, intrinsic IEC measurement, pollutant-specific testing under clearly defined conditions, and dynamic evaluation where continuous operation is intended. Regeneration studies should report not merely removal efficiency after several cycles but also capacity retention, ligand leaching, mass loss, swelling changes, mechanical damage, and alteration of surface chemistry. Four to six cycles, as reported for several GMA-based systems [109,110,112,113], demonstrate short-term reuse but cannot establish long-term operational durability. Furthermore, comparisons among studies remain difficult because IEC, adsorption capacity, water uptake, and dynamic capacity are reported using different mass bases, counterion forms, pH values, and flow conditions. Future studies should therefore adopt standardized reporting, characterize materials in their hydrated operational state, and distinguish clearly among chemical conversion, total functionality, accessible capacity, and dynamic utilization [114,115,116]. Such an integrated framework would make it possible to connect synthesis and functionalization with experimentally verified structure and, ultimately, with credible performance in water treatment and resource recovery.

## 5. Ion-Exchange, Adsorption, and Mass-Transfer Mechanisms

Ion uptake by epoxy-based materials is governed by electrostatic attraction, ion exchange, coordination, and chelation, often acting simultaneously (Table 2). The dominant interaction depends on ligand chemistry and contaminant speciation, but the strength of mechanistic evidence varies considerably. Sulfonated polymers have been associated with oxyanion retention and Cu(II) exchange, whereas amine-functionalized materials favour metal coordination through nitrogen donors [47,48,50,65,66]. However, these assignments often rely on FTIR spectra and adsorption behaviour rather than direct evidence of counter-ion displacement. The distinction becomes less clear for Pt(IV) and Pd(II) chloro-complexes, which can interact with protonated amines through electrostatic attraction alongside possible coordination [101]. Polyaminophosphonate-functionalized PGMA offers a stronger basis for multidentate metal binding, supported by XPS and proton-release measurements, although exchange stoichiometry remains unresolved [112]. A comparison of more recent systems exposes the same uncertainty. Increased Cu(II) uptake by PGMA–EDA at higher pH is consistent with greater availability of unprotonated amines, whereas Co(II) binding by aminated fibres is supported mainly by indirect structural characterization [117]. Co(II) adsorption by phosphoric acid- and amidoxime-functionalized polypropylene fibres was supported by FTIR and SEM characterization, although the specific metal–ligand binding interactions were not directly confirmed [118]. Thiourea-functionalized resins provide more direct spectroscopic evidence of Mo(VI) and W(VI) interactions with sulfur- and nitrogen-containing groups, but their binding geometry remains unconfirmed [119]. Conversely, proton release during arsenic uptake by epoxy-crosslinked guar gum cannot distinguish ion exchange from ligand-assisted complexation [120]. The critical limitation is therefore not the absence of plausible binding mechanisms, but the frequent inability to discriminate among them experimentally. Such an integrated framework connects material design, functionalization, accessible capacity, water-matrix effects, and operational performance in water treatment and resource recovery (Figure 4).

Solution chemistry further determines whether binding sites are accessible and selective. At low pH, protonated amines favour anion attraction but generally suppress coordination with metal cations. At higher pH, reduced protonation can improve metal binding, although hydrolysis and precipitation must also be considered. These effects complicate comparisons of adsorption capacities measured under different experimental conditions (Table 3). Beyond these effects, ion-imprinted materials introduce recognition sites designed to favour particular ions. As a comparative example outside the epoxy/GMA-based material class, La(III)-imprinted chitosan hydrogels showed enhanced recognition relative to non-imprinted controls, but their precise coordination geometry remained unresolved [121]. Within the scope of this review, chitosan–GMA/bentonite composites retained Pb(II) and Cd(II), although their reported capacities could not be directly related to measured ligand densities [122]. Another non-epoxy/GMA comparative example, As(V)-imprinted diatomite showed stronger discrimination against Cr(VI) and Mo(VI), suggesting that cavity geometry can contribute beyond general electrostatic attraction [123]. However, apparent selectivity also depends on competing-ion speciation and experimental conditions (Table 4). DEAE-functionalized PGMA illustrates this dependence because Pd(II) binding may shift between chloro-complex attraction and nitrogen coordination as solution chemistry changes [124]. Au(III) uptake by EDA-functionalized PGMA introduces another complication, as partial reduction can occur alongside adsorption [125]. Consequently, high uptake does not necessarily indicate stronger coordination or greater exchange-site utilization.

Functional-group density provides a useful starting point for interpreting adsorption capacity, but it is not equivalent to the number of accessible binding sites. PEI-functionalized magnetic microspheres exhibited a fitted Ce(III) capacity of 179.53 mg g^−1^, although the density of available coordinating groups was not quantified [126]. Likewise, DTPA-functionalized polyHIPE beads demonstrated multimetal uptake, but missing ligand-density and metal-specific capacity data prevented evaluation of binding-site utilization [127]. More informative comparisons are possible when functional-group loading is measured independently. CSSNa-functionalized magnetic PGMA contained 1.6747 mmol g^−1^ of dithiocarbamate groups and exhibited fitted capacities of 229.41 and 210.65 mg g^−1^ for Pb(II) and Cd(II), respectively [128]. Nevertheless, the proposed coordination stoichiometry was not directly verified. A similar limitation arises in DETA-functionalized cellulose, where an amino-group density of 10.26 mmol g^−1^ exceeded the Ni(II) uptake of approximately 4.84 mmol g^−1^ [129]. This difference indicates that not all measured amine groups contribute independently to metal binding. Steric restrictions, protonation, hydration, and multidentate coordination may reduce effective site utilization. Moreover, Langmuir-derived maximum capacities cannot independently establish binding-site density or molecular stoichiometry. Therefore, functional-group loading, accessible ion-exchange capacity, and contaminant-specific uptake should be reported separately.

**Table 2:** Functional Groups, Binding Mechanisms, and Supporting Evidence.

Ref.	Material and Active Groups	Target Contaminant	Functional-Group Density or IEC	Proposed Binding Mechanism	Supporting Evidence and Mechanistic Limitations
[117]	PGMA–EDA beads; primary amines; N = 4.006 wt%	Cu(II), SMZ	NR	Cu–N coordination; proton displacement	pH changes, FTIR, elemental analysis; no XPS or counter-ion balance
[48]	MB–SPGMA nanoparticles; sulfonate and immobilized MB	Cr(VI), Mn(VII)	NR	Electrostatic attraction; proposed oxyanion exchange	FTIR, SO_3_ bands at 1050–1060 cm^−1^; no counter-ion balance or post-adsorption XPS
[65]	GMA–ACN–PEI; primary/tertiary amines	Cu(II), Pb(II)	SEC = 6.22 meq g^−1^	Ion exchange and N coordination	FTIR, titrimetric SEC; no XPS or counter-ion balance
[47]	MB–SPGMA-co-PMMA; –SO_3_H, –OH, epoxide, MB	Cr(VI), Mn(VII)	NR	Electrostatic association; proposed counter-ion displacement	FTIR; no IEC, XPS or counter-ion balance
[118]	PP-g-(AN-co-AAc), modified with phosphoric acid and amidoxime groups; carboxyl, phosphorus-containing and amidoxime functionalities	Co(II)	NR	pH-dependent adsorption attributed to reduced electrostatic repulsion at higher pH and interactions with oxygen- and nitrogen-containing functional groups;	FTIR confirmed functionalization; SEM showed increased fiber diameter (24–29 to 40–47 µm). Phosphoric acid addition improved adsorption. No quantitative binding-site density, XPS evidence, metal–ligand coordination analysis, or direct mechanistic verification reported.
[119]	Thiourea–TD301; C=S, –NH_2_; N = 9.52 at%, S = 5.90 at%	Mo(VI), W(VI)	NR	S/N coordination and electrostatic attraction	Post-adsorption XPS, FTIR, zeta potential; binding stoichiometry unconfirmed
[120]	Epoxy-crosslinked guar gum; cis-diol groups	As, Sb	CEC = 2.5 meq g^−1^	Proposed cis-diol coordination; proton release	pH decrease, FTIR, TGA; no direct coordination or exchange-stoichiometry evidence
[50]	PES-g-GMA–SO_3_H membrane; grafting 9–41%	Cu(II)	IEC NR	Proposed sulfonate-mediated cation exchange	FTIR, grafting analysis; no counter-ion balance; source–data correspondence needs verification
[101]	Super-amino PGMA microspheres; –NH_2_, –NH–	Pt(IV), Pd(II)	NR	Chloro-complex attraction and N coordination	XPS, FTIR, zeta potential, XRD, EDS; contributions not separated
[122]	COS-g-GMA/PPG/bentonite; –NH_2_, –OH, silicate	Pb(II), Cd(II)	NR	N/O coordination	FTIR, SEM–EDX, XRD, EDTA desorption; no XPS or counter-ion balance
[112]	Polyaminophosphonate–PGMA; P/N donor groups	Y(III), La(III)	NR	P/N chelation; proton displacement	XPS, FTIR, pH titration, modelling; no counter-ion balance
[124]	PGMA–DEAE macroporous spheres; tertiary amines	Pd(II)	NR	[PdCl_4_]^2−^ attraction in acid; N coordination at higher pH	XPS, zeta potential; mechanism depends on Pd speciation
[125]	EDA–poly(GMA-co-EGDMA); amine/hydroxyl groups	Au(III)	NR	[AuCl_4_]^−^ attraction, N/O coordination, partial Au(III) reduction	FTIR, XPS; reduction complicates exchange stoichiometry
[126]	Magnetic PMMA–PGMA–PEI; –NH_2_, –NH–	Ce(III)	NR	Proposed Ce–amine coordination	FTIR confirms amination; no post-adsorption XPS
[127]	DTPA-functionalized epoxy polyHIPE beads; carboxylate/N donors	Cu, Cr, Pb, Fe, Cd	NR	Proposed multidentate DTPA chelation	Multimetal removal; no direct post-adsorption spectroscopy
[66]	EDA–PGMA-grafted PS; –NH_2_, –NH–	Cu(II), Cd(II)	NR	Amine coordination	FTIR, SEM, elemental analysis, XRD; no XPS or counter-ion balance
[128]	Fe_3_O_4_-PGMA-g-TETA-CSSNa; S/N/O donors	Pb(II), Cd(II)	CSS = 1.6747 mmol g^−1^	Multidentate coordination; electrostatic contribution	Post-adsorption XPS; proposed 2:1 binding structure unconfirmed
[129]	GMA-grafted cellulose–DETA; amines	Ni(II)	Amines = 10.26 mmol g^−1^	Multidentate Ni–N chelation	Post-adsorption XPS, FTIR shifts; site accessibility unquantified
[130]	Amino-functionalized PGMA fixed-bed sorbent	Vanadium oxyanions	NR	Proposed protonated-amine attraction/anion exchange	No post-adsorption spectroscopy, IEC or counter-ion balance; V speciation NR
[131]	Magnetic poly(AMA-co-GMA); –SH, –NH_2_, –OH	Pb, Hg, Cd, Cu, Zn, Ni	NR	Proposed thiol–metal coordination with N/O contributions	EDS and HSAB rationale; no post-adsorption S 2p XPS or EXAFS
[132]	GMA-grafted sepiolite–PO_3_H_2_; grafting 479%	Cu(II)	NR	Phosphonate–Cu coordination; proposed proton exchange	FTIR confirms modification; no post-adsorption XPS or counter-ion balance
[133]	Magnetic P(GMA-co-DVB) functionalized with EDA, DETA, or TETA; amine and hydroxyl groups.	Cr(VI) oxyanions	NR	Electrostatic attraction between protonated amines and Cr(VI) oxyanions; possible anion exchange.	FTIR and elemental analysis confirmed functionalization; optimal adsorption at pH 2 (DETA: 98.4% removal). Steric hindrance limits site accessibility; direct evidence of ion exchange is lacking.
[134]	Hydroxylamine–PET-g-GMA; –NHOH; N = 5.99%	Cu(II), Ni(II), Cd(II)	NR	Proposed N,O-bidentate coordination	Elemental analysis, competition; no XPS, FTIR or binding stoichiometry
[135]	PGMA–EDA–Fe(III) gel; amine/Fe sites	As(III), As(V)	Amine content measured; IEC NR	Fe-associated arsenic binding	Functional-group and swelling analyses; binding structure unconfirmed
[136]	RAFT-mediated GMA-grafted PP/PE/cellulose	Phosphate	NR	Proposed binding through functionalized grafts	ATR-FTIR, XRF, SEM; direct adsorption-site evidence limited
[137]	Crosslinked epoxy–PEI/BTA capsules; amines	Methyl orange, Cr(VI), phosphate	NR	Swelling-mediated uptake; proposed amine–anion association	FTIR, XPS, SEM; contaminant-specific binding stoichiometry NR
[138]	Radiation-grafted PE–GMA/acrylic acid; –COOH	MB, crystal violet, Cu(II), Pb(II)	NR	Carboxyl-associated electrostatic binding/coordination	FTIR, SEM; no counter-ion balance
[139]	Epoxy-functionalized Trichosanthes dioica seed coat; cationic sites	Acid Red 18	NR	Electrostatic dye attraction	FTIR, SEM–EDS; no quantitative exchange-site analysis
[140]	Magnetic jute cellulose/Fe_3_O_4_/P(MAA–GMA); –COOH	Methylene blue	NR	Carboxylate–cationic dye association	XPS, BET/BJH; exchange stoichiometry unconfirmed
[141]	Tannic-acid-functionalized epoxy network; phenolic –OH	Methylene blue	NR	Proposed pH-dependent electrostatic/H-bond interactions	Structural and porosity analyses; no direct binding-site quantification
[142]	GMA-grafted Teflon; phosphoryl groups	U(VI)	NR	Phosphoryl–U coordination	XPS; exchange stoichiometry NR
[143]	5-ASA–poly(GMA-co-EGDMA); acidic/N-containing groups	Cr(VI)	Epoxide = 2.87–5.02 mmol g^−1^; acid number = 14.0–30.9 mg KOH g^−1^	Proposed protonated-site/oxyanion attraction	FTIR, TG–FTIR; epoxide density is not IEC
[144]	Starch/PVA–GMA–IDA hydrogel; IDA sites	Cu(II)	NR	IDA–Cu chelation	Structural characterization; binding-site density NR
[145]	PGMA-grafted nanocellulose/nanobentonite–PEI; amines	Methyl orange, eosin yellow	NR	Protonated-amine/anionic dye attraction	Structural analyses; no counter-ion balance
[146]	Crosslinked PGMA–PEI; protonated amines	Acid Red 27, nitrite, acetaminophen	Precursor epoxide = 4.83 ± 0.02 mmol g^−1^; IEC NR	Electrostatic anion binding	Polymer characterization; precursor epoxide density is not final IEC
[147]	Magnetic wood fibers coated with pGMA, pAA or pHEMA	MB, RhB	NR	Polymer-dependent dye adsorption	FTIR, TG, SEM; individual binding contributions unresolved
[148]	Bombax ceiba gum–GMA–cysteine network; N/S/O sites	Crystal violet, MB, methyl orange	NR	Proposed electrostatic and functional-group interactions	FTIR, XRD, FESEM–EDS; no direct binding stoichiometry
[149]	GMA/TMPTMA copolymer; amine groups	Pb(II), Co(II), Cd(II), Cr(III)	NR	Proposed amine–metal binding	FTIR, Raman, solid-state NMR, BET; direct metal coordination evidence limited
[150]	Cotton-g-GMA–phosphoric acid; phosphate groups	Metformin hydrochloride	NR	Proposed phosphate-mediated electrostatic binding	FTIR, SEM, XRD, surface charge; no exchange stoichiometry
[151]	Magnetic GO-g-GMA–β-cyclodextrin; host cavities	Gemcitabine	NR	Proposed β-CD host–guest inclusion	Epoxy ring-opening attachment; inclusion complex not directly quantified
[152]	Cellulose-g-GMA–hyperbranched PEI; amines; pH_p_zc = 7.7	Tartrazine, amaranth	NR; graft yield = 89.28%	Protonated-amine/anionic dye attraction	FTIR, ^13^C NMR, BET; graft yield is not IEC
[153]	PGMA-coated satsuma mandarin peel; hydroxylamine/HCl-modified groups	Cr(VI)	NR	Reduction-coupled adsorption	FTIR, SEM–EDS; dissolved Cr(III) detected; uptake and reduction not separated
[154]	Magnetic α-cellulose/Fe_3_O_4_; pGMA, pAA, pHEMA coatings	MB, RhB	NR	Polymer-dependent dye binding	FTIR, SEM, elemental mapping; site-specific evidence limited
[155]	Magnetic PS/PVBC-g-PGMA–2M12C4; crown ether	Li(I)	NR	Li–crown ether host–guest recognition	Crown-ether functionalization and adsorption selectivity; binding stoichiometry NR
[156]	Poly(HEMA-GMA) membrane–DFO; chelator groups	Fe(III)	NR	Fe–DFO chelation	ATR-FTIR, elemental analysis; DFO loading NR
[157]	Nylon-6-g-GMA–DAP/DETA; amines	Diclofenac sodium	DAP = 3.39; DETA = 2.66 mmol g^−1^	Electrostatic and H-bond interactions	FTIR, XPS, SEM; no quantitative exchange stoichiometry
[158]	Mo-imprinted epoxy/PEG-600/hyperbranched polyamide-amine	Mo(VI)	NR	Imprint-directed Mo recognition	FTIR, XPS, SEM–EDS; coordination geometry NR
[159]	Chitosan–GMA hydrogels; mono-/disulfonated groups	Cu(II), ciprofloxacin	Sulfonic substitution = 0.74/1.58 mmol g^−1^; GMA substitution = 3.39/2.21 mmol g^−1^	Proposed ionic and coordination interactions	NMR, FTIR; substitution degrees are not measured IEC
[160]	PET-g-GMA–IDA/NaOH/CS_2_; carboxylate/S groups	Pb(II)	NR	Proposed Pb coordination/ion exchange	FTIR, SEM, DSC; no direct metal-binding spectroscopy
[161]	Loofah-g-PGMA–DETA/PEI; protonated amines	Cr(VI)	NR	Electrostatic attraction, proposed exchange and Cr(VI) reduction	FTIR, EDS; reduction and adsorption not separated
[162]	NWPP-g-GMA–DETA–phosphorylated groups; N/P donors	La(III)	NR	Proposed La binding at N/P sites	FTIR, SEM, XRD; post-adsorption coordination evidence limited
[163]	D314-g-PGMA–SEA; sulfonic groups	Ga(III)	NR	Proposed functional-group-mediated Ga binding	FTIR; no direct binding stoichiometry
[164]	PP-g-GMA–NMDG; imprinted borate-recognition sites	Borate	NR; grafting = 203.26%	Imprint-assisted NMDG–borate recognition	FTIR, XRD, SEM; grafting percentage is not IEC
[165]	Fe_3_O_4_/chitosan-g-PGMA–HYD/IMDZ; N donors	U(VI)	NR	Proposed N-donor–U coordination	Functionalization and uptake evidence; binding geometry NR
[166]	Fe_3_O_4_@SiO_2_@GMA–CA–SH; thiol groups	As(III)	NR	As–S affinity/coordination	Pre-/post-adsorption XPS; thiol-site density NR
[167]	Magnetic PGMA–hyperbranched polyglycerol–COOH	MB, rhodamine 6G	NR	Carboxylate–cationic dye association	Functionalization and uptake evidence; exchange stoichiometry NR
[168]	Amine-cured epoxy networks E1–E4	Acridine orange	NR	Proposed dye–polymer interactions	FTIR, TGA; ion exchange not independently established
[169]	Chitosan/rice-straw cellulose-g-GMA; amino/hydroxyl groups	Cd(II)	NR	Proposed N/O coordination	FTIR, XRD, SEM; no post-adsorption XPS
[170]	PGMA–EDA/PDA/PEI; amines	Glyphosate	Amine loading measured; IEC NR	Electrostatic attraction and H-bonding	FTIR, XPS, elemental analysis; exchange stoichiometry NR
[171]	Poly(NaSS-co-GMA) foams; –SO_3_^−^; NaSS = 43/57/68 mol%	Methylene blue	IEC NR	Sulfonate ion pairing; aromatic–aromatic interactions	Composition and adsorption analysis; NaSS fraction is not IEC
[49]	Crystal-violet-loaded SPGMA nanoparticles	Cr(VI)/dichromate	NR	Proposed dye-mediated dichromate association	FTIR, SEM–EDAX; immobilized-dye stability and exchange stoichiometry unresolved
[172]	Sulfonated PGMA nanoparticles; –SO_3_^−^	Crystal violet	NR	Sulfonate–cationic dye association	FTIR, SEM–EDAX; no counter-ion balance
[173]	PEHA@GMA-DVB ± Fe_3_O_4_; polyamines	Cr(VI)	NR	Proposed protonated-amine/Cr(VI) association	Isotherm/D–R analyses; no direct exchange stoichiometry
[174]	GMA-grafted chitosan functionalized with 1,4-bis(3-aminopropyl)piperazine (CH-ANP); amino, hydroxyl, and nitrogen-containing groups	Cu(II), Cd(II), Pb(II)	N content: 5.20 mmol/g (CH-ANP) versus 5.27 mmol/g (pristine chitosan); IEC not reported	Metal coordination with nitrogen-containing Lewis-basic groups; binding geometry unconfirmed	FTIR, ^13^C NMR, and elemental analysis confirmed functionalization; XRD and TGA characterized structural changes. No direct metal–ligand evidence or accessible-site quantification.

NR = not reported; IEC = ion-exchange capacity; SEC = sorption/exchange capacity as designated in the source; CEC = cation-exchange capacity; MB = methylene blue; SMZ = sulfamethazine. Functional-group densities are reported in mmol g^−1^ unless otherwise indicated. Proposed mechanisms are distinguished from direct spectroscopic evidence.

**Table 3:** Adsorption Capacity, Equilibrium, and Mass-Transfer Characteristics.

Ref.	Material and Target	Experimental Conditions	Equilibrium Uptake or Maximum Capacity	Isotherm Model and Affinity Parameters	Kinetics and Equilibrium Time	Mass-Transfer Characteristics
[117]	PGMA–EDA; Cu(II) ± SMZ	pH 1/5; 25 ± 1 °C; C_0_ = 1–5 mmol L^−1^	q_m__a__x_ = 0.244/0.794 mmol g^−1^ at pH 1/5	Langmuir; K^f^ = 0.155/0.573 at pH 1/5	PSO; k_2_ = 0.324/0.0264 g mmol^−1^ min^−1^ at pH 1/5; equilibrium ≈12 h	Diffusion parameters NR
[48]	MB–SPGMA; Cr(VI), Mn(VII)	C_0_ = 2–8 mg L^−1^; 25–60 °C	Combined experimental uptake = 27.32 mg g^−1^	Freundlich; constants NR	PSO; rate constants/time NR	NR
[65]	GMA–ACN–PEI; Pb(II), Cu(II)	NR	q_m__a__x_ NR	Langmuir; R^2^ = 0.9979/0.9857 for Pb/Cu; K_l_ NR	NR	NR
[47]	MB–SPGMA-co-PMMA; Cr(VI), Mn(VII)	pH 7–8; room temperature; C_0_ = 2–8 mg L^−1^	q_e_ = 0.57/0.88 mg g^−1^ for Cr/Mn at C_0_ = 8 mg L^−1^	NR	Equilibrium ≈3 h; kinetic constants NR	NR
[118]	PP-g-(AN-co-AAc), modified with phosphoric acid and amidoxime groups	initial Co(II) ≈50 mg/L; solution volume 40 mL; pH 6.5; 25–65 °C; adsorbent mass 0.005–0.1 g; contact time 2–24 h	99.78% removal at 25 °C; 99.96% at 55 °C.	NR	Rapid initial uptake; apparent equilibrium after approximately 4 h; no kinetic rate constants or model fitting reported	nitial rapid adsorption followed by slower uptake attributed to decreasing availability of vacant sites
[119]	Thiourea–TD301; Mo(VI), W(VI)	pH 1.5/0.5; 298 K; C_0_ = 100 mg L^−1^	NR	NR	Fast uptake reported; numerical parameters NR	NR
[120]	Epoxy–guar gum; As, Sb	pH decreased from 10 to 5	As uptake = 29.445 mg g^−1^	NR	NR	NR
[50]	PES-g-GMA–SO_3_H; Cu(II)	35 °C; C_0_ = 5–100 mg L^−1^; grafting 9–41%	q_m__a__x_ = 111/149 mg g^−1^ at 9/25% grafting; dynamic capacity = 64.05 mg g^−1^	NR	NR	Grafted-layer diffusion proposed as limiting; D_e_, D_p_ NR
[101]	Super-amino PGMA; Pt(IV), Pd(II)	pH ≈4; 298 K	Uptake = 3.93/4.99 mmol g^−1^ for Pt/Pd	NR	Equilibrium 15 min	NR
[122]	COS-g-GMA/PPG/bentonite; Pb(II), Cd(II)	pH ≈5–6	Reported capacities = 177/158 mg g^−1^ for Pb/Cd	Freundlich; constants NR	PSO; R^2^ = 0.9994/0.9984 for Pb/Cd	NR
[112]	Polyaminophosphonate–PGMA; Y(III), La(III)	Initial pH 5.0	q_m__a__x_ = 0.744/0.795 mmol g^−1^ for Y/La	Langmuir; constants NR	PSO; t_1_/_2_ = 29.3/16.2 min for Y/La; equilibrium ≈180–240 min	Vermeulen model; kբ, D_e_, D_p_ NR
[124]	PGMA–DEAE; Pd(II)	pH 4–6 or concentrated HCl; 298 K	q_e_ = 1.22 mmol g^−1^	NR	Rapid uptake; numerical constants NR	NR
[125]	EDA–poly(GMA-co-EGDMA); Au(III)	HCl medium; room temperature	q_e_ = 187.22 mg g^−1^	NR	PSO/Avrami; t_1_/_2_ = 5 min; equilibrium 30 min	Boyd and three-stage IPD; kբ, D_e_ NR
[126]	Magnetic PMMA–PGMA–PEI; Ce(III)	pH 6; 298.15–318.15 K; C_0_ = 50–450 mg L^−1^	q_m__a__x_ = 179.53; experimental q_e_ = 142.69 mg g^−1^	Langmuir; K_l_ = 0.1396 L mg^−1^ at 298 K	PSO; k_2_ = 4.76 × 10^−5^ g mg^−1^ min^−1^; equilibrium 360 min	NR
[127]	DTPA–epoxy polyHIPE; Cu, Cr, Pb, Fe, Cd	Multimetal trace solution	Numerical uptake NR	Freundlich; constants NR	NR	NR
[66]	EDA–PGMA-g-PS; Cu(II), Cd(II)	pH 3	q_m__a__x_ = 1.19/1.95 mmol g^−1^ for Cu/Cd	Langmuir; constants NR	PSO; R^2^ = 0.9994/0.9984 for Cu/Cd	NR
[128]	Fe_3_O_4_-PGMA-g-TETA-CSSNa; Pb(II), Cd(II)	pH 4–8; 293 K	q_m__a__x_ = 229.41/210.65 mg g^−1^ for Pb/Cd	Langmuir; constants NR	PSO; equilibrium 10 min; E_a_ = 31.69/16.84 kJ mol^−1^ for Pb/Cd	Diffusion coefficients NR
[129]	Cellulose-g-GMA–DETA; Ni(II)	pH 5; 50 °C	q_m__a__x_ = 284 mg g^−1^; comparator = 188 mg g^−1^	Langmuir; constants NR	PSO; time and constants NR	NR
[130]	Amino-PGMA fixed bed; vanadium oxyanions	C_0_ = 50–100 mg L^−1^; bed 2–4 cm; flow 7.5–12.5 mL min^−1^	Maximum column capacity = 75.5 mg g^−1^	Batch isotherm NR	Thomas, Yoon–Nelson, BDST; R^2^ ≥ 0.90	Column breakthrough modelling; film/pore contributions unresolved
[131]	Magnetic poly(AMA-co-GMA); Pb, Hg, Cd, Cu, Zn, Ni	NR	Numerical q_m__a__x_ NR; Pb/Hg highest	Langmuir; constants NR	PSO; constants NR	NR
[132]	Phosphonate–GMA–sepiolite; Cu(II)	pH, C_0_ NR	q_m__a__x_ = 134.5 mg g^−1^	Langmuir; constants NR	PSO; constants/time NR	NR
[134]	Hydroxylamine–PET-g-GMA; Cu(II), Ni(II), Cd(II)	pH 6; 75 °C; C_0_ = 300 mg L^−1^	Uptake = 27.76/63.86/66.73 mg g^−1^ for Cu/Ni/Cd	NR	Equilibrium = 90/45/120 min for Cu/Ni/Cd	NR
[135]	PGMA–EDA–Fe(III) gel; As(III), As(V)	Groundwater; near-neutral pH	Saturation uptake = 31.0/39.0 mg g^−1^ for As(III)/As(V)	NR	Rapid uptake; constants/time NR	NR
[136]	RAFT GMA-grafted PP/PE/cellulose; phosphate	Aqueous batch; other conditions NR	Improved uptake; numerical capacity NR	NR	NR	Grafting/accessibility studied; transport coefficients NR
[137]	Epoxy–PEI/BTA capsules; MO, dichromate, phosphate	pH 3; 3.5 wt% NaCl; 60 °C	Reported capacity = 83.53 mg g^−1^; analyte assignment unverified	NR	Swelling/release depend on EP:PEI ratio	Swelling-mediated transport; coefficients NR
[138]	PE–GMA/acrylic acid; MB, CV, Cu(II), Pb(II)	25 °C	Adsorption capacities reported in the original study; numerical values not independently verified.	Isotherms evaluated; constants NR	PFO/PSO evaluated; constants NR	NR
[139]	Epoxy-modified seed coat; Acid Red 18	Aqueous batch; pH/C_0_ NR	q_m__a__x_ = 166.66/200 mg g^−1^ for raw/modified	Langmuir; constants NR	PSO; constants NR	NR
[140]	Jute cellulose/Fe_3_O_4_/P(MAA–GMA); MB	pH 8	q_m__a__x_ = 181 mg g^−1^	Langmuir; R^2^ = 0.9998	PFO preferred over PSO; constants/time NR	NR
[141]	Tannic-acid epoxy; MB	Near-neutral pH; real wastewater	q_m__a__x_ ≈8.0 mg g^−1^; removal ≈95%	Model NR	PSO; constants NR	Mesoporous structure; quantitative transport NR
[142]	Phosphate–GMA/Teflon; U(VI)	Neutral pH; batch/column	Maximum uptake = 72.4 mg g^−1^	Langmuir; constants NR	PSO; constants/time NR	Column tested; breakthrough parameters NR
[143]	ASA–poly(GMA-co-EGDMA); Cr(VI)	Acidic pH	Uptake ≈100 mg g^−1^	NR	NR	Surface area decreased 151→39 m^2^ g^−1^ with increasing GMA
[144]	Starch/PVA–GMA–IDA; Cu(II)	pH 5; 298 K; multimetal solution	q_m__a__x_ = 285.7 mg g^−1^	Langmuir; R^2^ = 0.995	PSO; R^2^ = 0.999	Diffusion contribution discussed; coefficients NR
[145]	PGMA–nanocellulose/bentonite–PEI; MO, EY	Acidic pH	q_m__a__x_ = 420.46/373.23 mg g^−1^ for MO/EY	Langmuir; constants NR	90% removal within 20 min; kinetic constants NR	NR
[146]	PGMA–PEI microspheres; Acid Red 27, nitrite, acetaminophen	Acidic/neutral pH	Uptake ≈ 0.25/0.023 mg g^−1^ for AR-27/nitrite; acetaminophen not removed	NR	PSO; rapid uptake; constants NR	Water uptake 80–120% at 40 min; diffusion coefficients NR
[147]	Magnetic wood fiber–pGMA/pAA/pHEMA; MB, RhB	MB C_0_ = 20; RhB C_0_ = 30 mg L^−1^; pH 7; 35 °C	pGMA–MB q_e_ = 16.15 mg g^−1^; pAA–RhB = 17.95 mg g^−1^	Langmuir for MB–pGMA	PSO; constants/time NR	NR
[148]	Bombax ceiba gum–GMA–cysteine; CV, MB, MO	Aqueous batch	CV q_m__a__x_ = 145.49 mg g^−1^	Langmuir; constants NR	PSO; R^2^ = 0.99141; contact 45 min	NR
[149]	GMA/TMPTMA amine resin; Pb(II)	Co/Cd/Cr comparisons; pH/C_0_ NR	Pb q_m__a__x_ = 316.45 mg g^−1^	Langmuir; constants NR	PSO; constants/time NR	NR
[150]	Cotton-g-GMA–phosphate; metformin	Aqueous batch	Maximum uptake = 24.7 mg g^−1^	Redlich–Peterson best fit; constants NR	PSO; constants/time NR	NR
[151]	Magnetic GO-g-GMA–β-CD; gemcitabine	pH 6.57; 25.40 °C; C_0_ = 1000 mg L^−1^	Optimized uptake = 844.51 mg g^−1^; not q_m__a__x_	NR	Optimized contact time 7.22 min	Intraparticle diffusion identified as main limitation
[152]	Cellulose-g-GMA–PEI; tartrazine, amaranth	C_0_ = 200 mg L^−1^; acidic pH favored	Uptake = 155.71/92.45 mg g^−1^ for tartrazine/amaranth	NR	80% removal within 5 min; equilibrium 15 min	BET area decreased 247.64→78.90 m^2^ g^−1^; diffusion coefficients NR
[153]	PGMA-coated mandarin peel; Cr(VI)/total Cr	Initial pH 1.4; 30–60 °C	Total Cr uptake = 110.23/219.28 mg g^−1^ at 30/60 °C	Langmuir/Jovanovic; constants NR	NR	NR; uptake includes reduction-related Cr speciation changes
[154]	Magnetic α-cellulose/Fe_3_O_4_–polymer coatings; MB, RhB	Aqueous; pH/C_0_ NR	Removal up to 91.36%/85.40% for MB/RhB; uptake NR	Isotherms assessed; parameters NR	Kinetic tests; parameters NR	NR
[155]	Magnetic PGMA–2M12C4; Li(I)	Aqueous brine-like mixtures	q_m__a__x_ = 10.62 mg g^−1^	Monolayer model; affinity constants NR	Equilibrium 120 min	Diffusion coefficients NR
[156]	Poly(HEMA-GMA)–DFO membrane; Fe(III)	Continuous-flow drinking water	Maximum continuous-system uptake = 25.7 mg g^−1^	NR	Continuous flow; kinetic constants NR	Breakthrough parameters NR
[157]	Nylon-6-g-GMA–DAP/DETA; diclofenac	Aqueous solution; pH/C_0_ NR	Removal = 99%/90% for DAP/DETA; uptake NR	NR	Rate constants/time NR	NR
[158]	Mo-imprinted epoxy/PEG/polyamide-amine; Mo(VI)	Metavanadate medium	q_m__a__x_ = 104.35 mg g^−1^	Model NR	PSO; constants/time NR	IPD constants NR
[159]	Chitosan–GMA sulfonated hydrogel; Cu(II), ciprofloxacin	pH 7	Removal = 83% Cu; 24–25% ciprofloxacin; uptake NR	NR	NR	Hydration/rheology assessed; diffusion parameters NR
[160]	PET-g-GMA–IDA/NaOH/CS_2_; Pb(II)	pH 5; 35 °C	Highest uptake = 94.14 mg g^−1^ for NaOH/CS_2_-treated PET	Langmuir; constants NR	Second-order model; constants/time NR	NR
[161]	Loofah-g-PGMA–DETA/PEI; Cr(VI)	318.15 K	q_m__a__x_ = 162.04/94.12 mg g^−1^ for DETA/PEI	Model NR	Numerical constants/time NR	NR
[162]	NWPP-g-GMA–DETA–phosphate; La(III)	Batch/fixed-bed; flow, bed height and C_0_ varied	Column capacity NR	NR	Bohart–Adams, Yan, log-Gompertz, Chu–Hashim models	Breakthrough modelling; numerical transport parameters NR
[163]	D314-g-PGMA–SEA; Ga(III)	pH 2; 25 °C	Equilibrium uptake = 288.81 mg g^−1^	Langmuir; constants NR	PFO; equilibrium 22 h	NR
[164]	PP-g-GMA–NMDG; borate	pH 9	Maximum uptake = 35.85 mg g^−1^	Freundlich; constants NR	PSO; 90% saturation within 60 min	NR
[165]	Fe_3_O_4_/chitosan-g-PGMA–HYD/IMDZ; U(VI)	Aqueous/seawater; other conditions NR	Uptake = 328.4/434.7 mg g^−1^ for HYD/IMDZ	NR	NR	NR
[166]	Fe_3_O_4_@SiO_2_@GMA–CA–SH; As(III)	Environmental water; 15 µg L^−1^ in pH tests	Quantitative extraction reported; adsorption capacity NR	NR	Contact 30 min in pH tests	NR
[167]	Magnetic PGMA–HPG–COOH; MB, R6G	MB optimum pH 7	q_m__a__x_ = 238.10/227.27 mg g^−1^ for MB/R6G	Langmuir; constants NR	PSO; 95% saturation within 10 min	NR
[168]	Amine-cured epoxy E1–E4; acridine orange	pH 10; 25 °C	q_m__a__x_ = 980.39/1515.2/1694.9/1851.9 mg g^−1^ for E1–E4	Langmuir; constants NR	Contact 60 min; kinetic constants NR	NR
[169]	Chitosan/rice-straw cellulose-g-GMA; Cd(II)	Aqueous batch; pH/C_0_ NR	q_e_ = 16.9 mg g^−1^	NR	PSO; R^2^ = 0.9979; equilibrium 10 min	NR
[170]	PGMA–EDA/PDA/PEI; glyphosate	Aqueous/mother liquor	PGMA–EDA q_m__a__x_ = 961.5 mg g^−1^	NR	PGMA–EDA equilibrium 5 min	NR
[171]	Poly(NaSS-co-GMA) foams; MB	Aqueous; NaSS = 43/57/68 mol%	Maximum uptake = 244/435/588 mg g^−1^	NR	PSO; k_2_ = 1.78/4.49/5.64 g mg^−1^ min^−1^	NR
[49]	CV-loaded SPGMA; Cr(VI)/dichromate	Aqueous; conditions NR	Uptake value NR in source extract	Isotherms evaluated; constants NR	Kinetic models evaluated; constants NR	NR
[172]	Sulfonated PGMA; crystal violet	Optimized batch conditions	Optimized uptake = 255.5 mg g^−1^	Freundlich; constants NR	Second-order kinetics; constants/time NR	Boyd model indicates film-diffusion control
[173]	PEHA@GMA-DVB ± Fe_3_O_4_; Cr(VI)	pH 2; electroplating wastewater	q_m__a__x_ = 192.31/217.39 mg g^−1^ for nonmagnetic/magnetic	Langmuir/D–R; reported adsorption energy = 25.01–31.62 kJ mol^−1^	Optimum contact 180 min	NR
[174]	GMA/piperazine-functionalized chitosan (CH-ANP); Cu(II), Cd(II), Pb(II)	Batch; 20 mg/25 mL; 0.7–2.0 mmol/L; neutral pH; 298 ± 1 K; 24 h	Uptake: Cu 2.82, Cd 1.96, Pb 1.60 mmol/g; Langmuir Ns: 2.94, 2.48, 1.85 mmol/g, respectively	Langmuir type I best fit; b (g/mmol): Cu 2.63, Cd 0.45, Pb 0.61; R^2^ ≥ 0.998	24 h equilibration; no fitted kinetic constants	Film/intraparticle diffusion and mass-transfer coefficients not evaluated

NR = not reported in the supplied table; PFO = pseudo-first-order; PSO = pseudo-second-order; IPD = intraparticle diffusion; MB = methylene blue; CV = crystal violet; MO = methyl orange; EY = eosin yellow; R6G = rhodamine 6G.

**Table 4:** Competitive Adsorption, Selectivity, Real-Water Performance, and Regeneration.

Ref.	Material and Target	Competing Ions or Contaminants	Selectivity and Competition Results	Real-Water or Column Performance	Regeneration and Reuse	Practical Limitations
[117]	PGMA–EDA; Cu(II)	Sulfamethazine (SMZ)	Cu uptake increased from 0.72 to 0.88 mmol g^−1^ with SMZ	NR	NR	Inorganic-ion competition and real-water validation NR
[48]	MB–SPGMA; Cr(VI), Mn(VII)	Cr(VI), Mn(VII) compared	Mn(VII) removal exceeded Cr(VI); selectivity coefficient NR	NR	NR	No controlled competition or quantitative selectivity
[65]	GMA–ACN–PEI; Pb(II), Cu(II)	Pb(II), Cu(II) compared	Pb(II) preferred over Cu(II); separation factor NR	NR	NR	Competitive adsorption conditions and quantitative selectivity NR
[47]	MB–SPGMA-co-PMMA; Cr(VI), Mn(VII)	Cr(VI), Mn(VII) compared	Mn(VII) uptake 0.88 vs Cr(VI) 0.57 mg g^−1^ at C_0_ = 8 mg L^−1^	NR	NR	Low uptake; binary competition not established
[118]	Phosphoric-acid/amidoxime-functionalized PP-g-(AN-co-AAc); Co(II), Zn(II), Ni(II), Cu(II)	Co(II), Zn(II), Ni(II), and Cu(II) examined individually	Single-ion removal: Co(II) 99.78%, Zn(II) 99.59%, Ni(II) 99.27%, Cu(II) 95.79%	Synthetic aqueous solutions only; no real-wastewater or continuous-flow column experiments	NR	No competitive adsorption, regeneration cycles, long-term stability, real-water validation, or scale-up assessment; γ-irradiation required for synthesis
[119]	Thiourea–TD301; Mo(VI), W(VI)	Mo(VI), W(VI)	Mo/W selectivity coefficient = 4.57 × 10^4^ at pH 1.5	NR	NR	Quantitative uptake capacity NR
[121]	La-imprinted chitosan hydrogel; La(III)	Competitor identities not clearly established	Selectivity coefficient = 2.04–3.51 relative to non-imprinted control	NR	NR	Competitor concentrations and selectivity basis require clarification
[101]	Super-amino PGMA; Pt(IV), Pd(II)	Co, Cu, Ni, Fe, Al, Cr	Pt/Pd selectivity maintained in presence of competing metals; coefficients NR	NR	NR	Competitive uptake and separation coefficients NR
[122]	COS-g-GMA/PPG/bentonite; Pb(II), Cd(II)	Pb(II), Cd(II) compared	Removal = 89% Pb and 78% Cd; binary competition NR	NR	EDTA-assisted desorption reported	Quantitative binary selectivity and cycle retention NR
[112]	Polyaminophosphonate–PGMA; Y(III), La(III)	Y(III), La(III)	La/Y selectivity coefficient ≈1.7	NR	NR	Broader competing-ion effects and regeneration NR
[124]	PGMA–DEAE; Pd(II)	Other metal ions; identities incompletely specified	Pd/metal separation ratio >30 to >100 near pH 4	NR	NR	Competitor concentrations and matrix effects incompletely specified
[127]	DTPA–epoxy polyHIPE; Cu, Cr, Pb, Fe, Cd	Cu, Cr, Pb, Fe, Cd in multimetal solution	Simultaneous removal demonstrated; quantitative selectivity NR	Multimetal trace solution tested	NR	Individual competitive capacities and real-water validation NR
[66]	EDA–PGMA-g-PS; Cu(II), Cd(II)	Na, K, Ca, Mg, Fe, Zn	Na/K/Ca/Mg had little effect; Fe/Zn caused slight inhibition; Cd uptake > Cu	NR	NR	Quantitative competition coefficients NR
[128]	Fe_3_O_4_-PGMA-g-TETA-CSSNa; Pb(II), Cd(II)	Cd, Pb, Cu, Ni	Selectivity order Cd > Pb > Cu > Ni	NR	NR	Separation coefficients and real-water performance NR
[130]	Amino-PGMA fixed bed; vanadium oxyanions	Competing ions NR	Selectivity NR	Fixed bed; maximum column capacity = 75.5 mg g^−1^; bed height 2–4 cm; flow 7.5–12.5 mL min^−1^	NR	No competitive breakthrough or regeneration data
[134]	Hydroxylamine–PET-g-GMA; Cu(II), Ni(II), Cd(II)	Cu(II), Ni(II), Cd(II)	Selectivity order Cd > Ni > Cu	NR	NR	Separation coefficients and real-water performance NR
[135]	PGMA–EDA–Fe(III) gel; As(III), As(V)	Saline constituents; identities NR	Saline tolerance and arsenic selectivity reported; coefficients NR	Groundwater tested	NR	Matrix composition and quantitative selectivity NR
[137]	Epoxy–PEI/BTA capsules; methyl orange, dichromate, phosphate	Multiple contaminants; 3.5 wt% NaCl	Multicontaminant adsorption reported; selectivity factors NR	Saline conditions tested; corrosion inhibition = 94.65% over 30 d	Swelling/release behavior investigated	Corrosion inhibition is not an adsorption removal metric
[138]	PE–GMA/acrylic acid; MB, crystal violet, Cu(II), Pb(II)	Four contaminants evaluated	Multicomponent selectivity NR	NR	Reuse evaluated; numerical retention NR	Competitive capacities and regeneration efficiency NR
[139]	Epoxy-modified seed coat; Acid Red 18	Competing solutes NR	NR	NR	Desorption >95% after 3 cycles	Single-dye tests; real-water performance NR
[141]	Tannic-acid epoxy; methylene blue	Wastewater constituents not specified	Selectivity coefficients NR	Real wastewater; ≈95% dye removal	Desorption >95% with organic eluent	Low reported capacity ≈8 mg g^−1^; eluent consumption
[142]	Phosphate–GMA/Teflon; U(VI)	Competing ions not quantified	NR	Batch and column tests reported	Nearly full capacity retained over 6 cycles	Competitive-ion selectivity and breakthrough parameters NR
[144]	Starch/PVA–GMA–IDA; Cu(II)	Other metal ions in multimetal solution	Separation factor up to 20.30	Multimetal solution tested	88.1% capacity retained after 6 cycles	Real-water performance NR; mechanical strength = 5.32 MPa
[145]	PGMA–nanocellulose/bentonite–PEI; methyl orange, eosin yellow	MO and EY investigated	Mixed-dye selectivity NR	NR	Multiple reuse cycles reported; retention NR	Real-water validation and quantitative regeneration NR
[147]	Magnetic wood fiber–pGMA/pAA/pHEMA; MB, RhB	MB and RhB evaluated using different polymer coatings	MB removal = 83.47% with pGMA; direct binary selectivity NR	NR	>70% removal after 5 cycles	Different adsorbents prevent direct selectivity comparison
[148]	Bombax ceiba gum–GMA–cysteine; CV, MB, MO	Crystal violet, methylene blue, methyl orange	At 45 min, removal = 93.36% CV, 83.68% MB, 3.89% MO; cationic dyes favored	NR	NR	Competitive mixed-dye tests and real-water validation NR
[149]	GMA/TMPTMA amine resin; Pb(II)	Co(II), Cd(II), Cr(III)	Pb preference reported; separation factors NR	NR	NR	Quantitative selectivity and real-water validation NR
[150]	Cotton-g-GMA–phosphate; metformin	Competing ions NR	NR	NR	≥5 cycles; ≈94.65% recovery in final cycle	Competitive-ion effects and real-water validation NR
[151]	Magnetic GO-g-GMA–β-CD; gemcitabine	Wastewater constituents not specified	Selectivity factors NR	Hospital wastewater; 94.2% removal	≈90% capacity retention after 6 cycles; ≈75% after 10	Competitive pharmaceuticals and matrix effects not quantified
[155]	Magnetic PGMA–2M12C4; Li(I)	Mg^2+^, Ca^2+^, Na^+^, K^+^	Li selectivity coefficient > 6.0	Brine-like mixtures tested	96.83% capacity retained after 8 cycles	Performance in authentic industrial brine not established
[156]	Poly(HEMA-GMA)–DFO membrane; Fe(III)	Competing metal ions	Selective Fe(III) removal; coefficients NR	Continuous-flow drinking-water treatment; uptake = 25.7 mg g^−1^	No significant capacity loss after 10 cycles	Quantitative competitive breakthrough parameters NR
[158]	Mo-imprinted epoxy/PEG/polyamide-amine; Mo(VI)	Vanadium species	Mo/V separation investigated; coefficient NR	Metavanadate medium tested	NR	Quantitative selectivity and regeneration NR
[161]	Loofah-g-PGMA–DETA/PEI; Cr(VI)	Other ions not specified	Selective Cr(VI) uptake reported; coefficients NR	NR	NR	Cr(VI) reduction complicates interpretation of selective adsorption
[162]	NWPP-g-GMA–DETA–phosphate; La(III)	Competing metals NR	NR	Fixed-bed tests; bed height, flow and inlet concentration varied; breakthrough models evaluated	NR	Numerical column capacity and multimetal selectivity NR
[164]	PP-g-GMA–NMDG; borate	SO_4_^2−^, Cl^−^, Na^+^, K^+^, Ca^2+^, Mg^2+^	Interfering-ion effects evaluated; quantitative selectivity NR	NR	78.1% efficiency retained after 5 cycles	Real-water validation and separation factors NR
[165]	Fe_3_O_4_/chitosan-g-PGMA–HYD/IMDZ; U(VI)	Seawater ions	Interference investigated; separation coefficients NR	Seawater tested	Magnetic recovery; cycle retention NR	Quantitative seawater selectivity and regeneration NR
[166]	Fe_3_O_4_@SiO_2_@GMA–CA–SH; As(III)	As(V) and interfering ions	As(III)/As(V) discrimination and ion tolerance investigated	Environmental-water extraction at trace concentrations	NR	Analytical extraction; bulk-treatment capacity NR
[167]	Magnetic PGMA–HPG–COOH; MB, R6G	MB and rhodamine 6G evaluated	Mixed-dye separation factors NR	NR	91.7% adsorption efficiency after 5 cycles	Competitive selectivity and real-water performance NR
[170]	PGMA–EDA/PDA/PEI; glyphosate	Mother-liquor constituents; identities NR	Competitive-ion coefficients NR	Glyphosate recovery from mother liquor investigated	Desorption and reuse reported; numerical retention NR	Matrix-specific selectivity and regeneration efficiency NR
[173]	PEHA@GMA-DVB ± Fe_3_O_4_; Cr(VI)	Electroplating wastewater constituents	Quantitative separation factors NR	Electroplating wastewater; removal = 76.42% nonmagnetic and 91.36% magnetic	Regeneration = 92–95%	Acidic optimum pH 2; competing-ion effects not isolated
[174]	GMA/piperazine-functionalized chitosan (CH-ANP); Cu(II), Cd(II), Pb(II)	Cu(II), Cd(II), Pb(II) tested separately; no mixed-metal experiments	Single-ion uptake: Cu (2.82) > Cd (1.96) > Pb (1.60 mmol/g); competitive selectivity not established	Synthetic solutions only; no column studies	NR	NR

NR = not reported in the supplied source table; MB = methylene blue; CV = crystal violet; MO = methyl orange; EY = eosin yellow; SMZ = sulfamethazine.

Adsorption rates are governed by the interplay between surface interactions and mass-transfer resistance, yet these contributions are rarely resolved independently (Table 3). Pseudo-second-order kinetics frequently describe uptake by functionalized epoxy polymers, but satisfactory fitting does not establish chemisorption or identify the rate-limiting step. Amino-functionalized PGMA achieved a maximum column capacity of 75.5 mg g^−1^ for vanadium, with breakthrough behaviour described using Thomas, Yoon–Nelson and BDST models [130]. Although useful for predicting column performance, these models do not independently distinguish external film resistance from intraparticle transport. Similar limitations apply to thiol-functionalized magnetic polymers and phosphonate-modified sepiolite, for which conventional kinetic fitting provides limited mechanistic resolution [131,132]. DETA-functionalized magnetic polymers offer a more detailed transport analysis, with Cr(VI) adsorption reaching apparent saturation within approximately 40 min and a Sips-fitted maximum capacity of 64.13 mg g^−1^ [133]. Intraparticle-diffusion and Boyd analyses suggested contributions from internal transport and liquid-film resistance, but neither mechanism was independently quantified. A complementary comparison emerges from hydroxylamine-functionalized PET, where Ni(II), Cu(II) and Cd(II) required 45, 90 and 120 min to reach equilibrium, respectively, with corresponding uptakes of 63.86, 27.76 and 66.73 mg g^−1^ [134]. These differences within the same polymer system reveal that faster equilibration does not necessarily correspond to greater uptake, while ion-specific coordination and aqueous speciation complicate attribution to diffusion alone. Thus, kinetic fitting, equilibrium time and column breakthrough provide complementary but non-equivalent information. Resolving the controlling transport processes requires independently determined diffusion coefficients, controlled hydrodynamic experiments and systematic variation of particle size or graft-layer thickness.

Beyond ligand chemistry, polymer architecture governs how contaminants reach and interact with functionalized surfaces. Grafting, crosslinking and swelling can alter hydration, pore accessibility and diffusion pathways, making structural effects difficult to separate from chemical affinity (Table 2 and Table 3). Fe(III)-modified PGMA–EDA gels achieved saturation capacities of 31 and 39 mg g^−1^ for As(III) and As(V), respectively, whereas RAFT-mediated GMA grafting onto polymeric and cellulosic substrates provided an alternative architecture for phosphate capture [135,136]. These systems demonstrate different functionalization strategies, but their structural contributions cannot be compared quantitatively without matched transport measurements. Swelling-dependent uptake in epoxy–PEI capsules further illustrates how polymer hydration can influence contaminant access, while radiation-grafted polyethylene and epoxy-modified biomass combine changes in surface chemistry with changes in substrate architecture [137,138,139]. The modified biomass increased Acid Red 18 capacity from 166.66 to 200 mg g^−1^, providing a direct within-study measure of functionalization benefit [139]. Structural effects become particularly apparent in dye adsorption. Magnetic jute composites exhibited a fitted methylene-blue capacity of 181 mg g^−1^, whereas tannic-acid epoxy networks reached only approximately 8 mg g^−1^ despite removing about 95% of the dye from real wastewater [140,141]. These results are not directly comparable because adsorption conditions and capacity definitions differ; importantly, high removal efficiency at a given contaminant loading does not imply high equilibrium capacity. Phosphoryl-functionalized Teflon achieved U(VI) uptake of 72.4 mg g^−1^, demonstrating that grafted architectures can also support metal capture [142]. A clearer structural trade-off emerged in 5-ASA-functionalized GMA copolymers, where increasing GMA content reduced BET surface area from 151 to 39 m^2^ g^−1^, while Cr(VI) uptake reached approximately 100 mg g^−1^ [143]. The surface-area decrease cannot by itself establish reduced aqueous accessibility, but it shows that greater incorporation of reactive monomers need not preserve the original porous structure. Polymer design must therefore balance chemical functionality with hydration, pore connectivity and transport accessibility rather than treating functionalization as an independent route to higher adsorption capacity.

Differences in adsorption capacity among nominally similar polymers reveal the importance of contaminant-specific interactions. IDA-functionalized hydrogels achieved a fitted Cu(II) capacity of 285.7 mg g^−1^, consistent with the effectiveness of multidentate ligands for metal retention [144]. For anionic dyes, however, electrostatic compatibility can be more influential than chelation. PEI-functionalized nanocellulose composites reached 420.46 and 373.23 mg g^−1^ for methyl orange and eosin yellow, respectively [145]. In sharp contrast, other PGMA–PEI microspheres adsorbed only approximately 0.25 mg g^−1^ of Acid Red 27 and 0.023 mg g^−1^ of nitrite, with negligible acetaminophen removal [146]. Although these materials share amine functionality, their markedly different performance cannot be explained by ligand identity alone. Polymer-coated magnetic fibres and cysteine-functionalized biopolymers reinforce this distinction, with adsorption varying according to surface chemistry and dye charge [147,148]. The latter removed 93.36% of crystal violet but only 3.89% of methyl orange under the reported conditions. Metal-binding GMA resins and phosphate-grafted cotton further demonstrate that the same epoxy platform can accommodate different interaction mechanisms, including metal coordination and ionic association with pharmaceutical compounds [149,150]. β-Cyclodextrin-functionalized magnetic composites introduce a further possibility through host–guest recognition, although their optimized gemcitabine uptake of 844.51 mg g^−1^ should not be interpreted as a fitted maximum capacity [151]. Meanwhile, hyperbranched PEI-functionalized cellulose retained anionic dyes despite a reduction in BET surface area from 247.64 to 78.90 m^2^ g^−1^ [152]. These contrasting results challenge simple structure–capacity correlations. Langmuir and Freundlich parameters are useful for describing equilibrium behaviour, but cross-material comparisons require consistent contaminant chemistry, experimental conditions and capacity definitions.

High adsorption capacity does not necessarily imply selective molecular recognition, particularly when contaminant removal involves chemical transformation (Table 4). Hydroxylamine-modified PGMA-coated biomass reached total chromium uptakes of 110.23 and 219.28 mg g^−1^ at 30 and 60 °C, respectively, but Cr(VI) reduction to Cr(III) complicates interpretation of the removal mechanism [153]. Polymer-dependent dye adsorption by magnetic cellulose composites presents a different limitation, as differences in removal efficiency do not independently establish competitive selectivity [154]. More convincing recognition strategies rely on defined ligand architectures. Crown-ether-functionalized PGMA achieved a Li(I) capacity of 10.62 mg g^−1^ and selectivity coefficients exceeding 6 against competing alkali and alkaline-earth ions [155]. DFO-functionalized membranes provided selective Fe(III) capture under continuous-flow drinking-water conditions, with a reported capacity of 25.7 mg g^−1^ [156]. Although these capacities are lower than those reported for many nonspecific adsorbents, their practical value lies in preferential recognition rather than maximum uptake alone. Aminated nylon membranes removed 99% and 90% of diclofenac using DAP and DETA functionalities, respectively, whereas Mo(VI)-imprinted epoxy materials achieved a capacity of 104.35 mg g^−1^ in vanadium-containing media [157,158]. The latter offers a more relevant competitive environment for assessing recognition, although quantitative separation factors remain unavailable. Sulfonated chitosan–GMA hydrogels removed approximately 83% of Cu(II) but only 24–25% of ciprofloxacin, illustrating contaminant-dependent uptake rather than demonstrated binary selectivity [159]. Modified PET and amine-functionalized loofah further extend metal and Cr(VI) adsorption, with the latter reaching 162.04 mg g^−1^ for DETA functionalization compared with 94.12 mg g^−1^ for PEI [160,161]. Such differences may reflect ligand architecture, accessibility or coupled redox reactions. Selectivity should therefore be distinguished from single-solute capacity, while adsorption and reduction must be quantified separately for redox-active contaminants.

The translation of batch adsorption performance into continuous-flow operation remains a major challenge for functionalized epoxy-based materials. Equilibrium capacity describes uptake under defined conditions, whereas dynamic performance additionally depends on contact time, mass-transfer resistance and breakthrough behaviour (Table 3 and Table 4). Phosphorylated GMA-grafted polypropylene was evaluated for La(III) removal in fixed beds using several breakthrough models, but neither numerical column capacity nor intrinsic diffusion coefficients were reported [162]. The distinction is particularly relevant to Ga(III) adsorption by D314-g-PGMA–SEA, which achieved an equilibrium uptake of 288.81 mg g^−1^ but required approximately 22 h to reach equilibrium [163]. Such slow uptake raises questions about performance at shorter residence times, without establishing diffusion as the controlling limitation. By comparison, NMDG-functionalized polypropylene reached 90% of its borate saturation uptake within 60 min, with a maximum capacity of 35.85 mg g^−1^ [164]. These systems cannot be ranked directly because their target ions and operating conditions differ, but they illustrate why capacity and uptake rate must be evaluated independently. Magnetic chitosan–PGMA composites achieved U(VI) uptakes of 328.4 and 434.7 mg g^−1^ with HYD and IMDZ functionalization, respectively, while thiol-functionalized magnetic materials were applied to trace As(III) extraction [165,166]. Their different applications highlight the distinction between bulk contaminant removal and analytical preconcentration, neither of which independently establishes continuous-flow performance. A similar caution applies to dye adsorbents. Carboxyl-functionalized magnetic PGMA reached fitted capacities of 238.10 and 227.27 mg g^−1^ for methylene blue and rhodamine 6G, respectively [167], whereas amine-cured epoxy networks yielded exceptionally high Langmuir capacities of 980.39–1851.9 mg g^−1^ for acridine orange [168]. These values require evaluation against the experimental concentration range, adsorption mass balance and capacity normalization before meaningful cross-material comparisons can be made. Conversely, GMA-grafted cellulose reached Cd(II) equilibrium within 10 min but exhibited a much lower uptake of 16.9 mg g^−1^ [169]. Collectively, these studies demonstrate that neither high fitted capacity nor rapid batch equilibration predicts column utilization. Establishing practical structure–performance relationships requires dynamic capacities measured at defined flow rates and bed depths, together with breakthrough profiles and independently assessed transport limitations.

Practical applicability depends not only on adsorption capacity but also on performance in complex water matrices, contaminant selectivity and sustained uptake over repeated treatment cycles (Table 4). Amine-functionalized PGMA exhibited a Langmuir-fitted glyphosate capacity of 961.5 mg g^−1^ and reached equilibrium within approximately 5 min, with recovery investigated in mother liquor [170]. Despite these favourable batch results, the absence of quantitative competitive adsorption data and multicycle capacity retention limits assessment of its operational robustness. Sulfonated GMA foams showed a systematic increase in methylene-blue uptake from 244 to 435 and 588 mg g^−1^ as the NaSS fraction increased from 43 to 57 and 68 mol%, respectively [171]. This composition-dependent improvement demonstrates enhanced uptake within a common material platform, but its persistence in chemically complex wastewater remains untested. For sulfonated PGMA nanoparticles, dye-mediated Cr(VI) capture introduces a different concern because the stability of immobilized crystal violet was not quantitatively established [49]. Related nanoparticles achieved an optimized crystal-violet uptake of 255.5 mg g^−1^, with Boyd analysis suggesting liquid-film resistance, indicating that favourable batch uptake does not eliminate potential transport limitations [172]. More direct evidence of wastewater applicability was obtained with magnetic polyamine-functionalized GMA, which removed 91.36% of Cr(VI) from electroplating wastewater compared with 76.42% for its nonmagnetic counterpart [173]. The 14.94-percentage-point improvement supports the benefit of magnetic modification under the tested conditions, although the optimum pH of 2 and unquantified matrix competition constrain extrapolation to other wastewater compositions. The reported 92–95% regeneration further indicates that adsorbed chromium could be released, but it should not be equated with retention of the original adsorption capacity over successive cycles. This distinction is important because desorption efficiency measures contaminant release, whereas capacity retention reflects the combined effects of incomplete regeneration, irreversible binding and material degradation. GMA- and piperazine-functionalized chitosan exhibited Cu(II), Cd(II) and Pb(II) uptake capacities of 2.82, 1.96 and 1.60 mmol g^−1^, respectively, but competitive adsorption, real-water performance and regeneration were not evaluated [174]. Across these studies, the strongest evidence concerns batch uptake and selected real-water removal tests, whereas competitive separation and sustained operation remain less comprehensively characterized. Practical validation therefore requires contaminant-specific mass balances, controlled matrix-interference experiments and adsorption-capacity retention measured over repeated cycles under representative treatment conditions.

A mechanistic understanding of epoxy-based ion-exchange materials requires connecting molecular binding to accessible capacity, transport and practical separation performance. These properties are related but cannot be inferred from one another. Spectroscopic evidence may support coordination without establishing exchange stoichiometry, while functional-group loading may exceed the number of sites available for adsorption. Likewise, equilibrium capacities and kinetic fits cannot independently identify the controlling transport process. The most informative studies combine complementary measurements, including chemical-state analysis, quantitative ligand characterization, competitive adsorption and regeneration. However, direct counter-ion balances, accessible IEC measurements, binding stoichiometry and intrinsic diffusion coefficients remain uncommon across the literature. The next step is not simply to maximize ligand density or fitted adsorption capacity. Instead, material development should establish how ligand architecture and polymer hydration influence site utilization, how transport resistance affects dynamic capacity, and whether selective uptake persists under realistic water chemistry. Quantitative competition tests, species-resolved mass balances and multicycle breakthrough experiments would provide the necessary links between molecular design and treatment performance. This integrated approach offers a stronger basis for evaluating epoxy-based adsorbents than isolated comparisons of maximum capacity or removal efficiency.

## 6. Applications in Water Treatment and Resource Recovery

Epoxy- and GMA-based materials have been investigated for the removal of toxic metal cations from municipal and industrial wastewater. Their practical applicability depends not only on maximum adsorption capacity but also on residual contaminant concentration, treatment time, competing ions, and performance in authentic wastewater. Aminated, polyamine-modified, thiol-containing, and aminocarboxylate-functionalized sorbents have been applied to Cu(II), Pb(II), Cd(II), Hg(II), Ni(II), and Co(II) removal [57,58,59,60,61,62,63,64,65,66]. Magnetic GMA-based resins offer additional advantages by facilitating the separation and recovery of fine adsorbent particles. For example, a magnetic PGMA resin demonstrated Hg(II) adsorption and was subsequently evaluated using wastewater rather than exclusively synthetic solutions [85]. Other reported systems include aminated PGMA beads for Cu(II) removal [117], and DTPA-functionalized polyHIPE beads for simultaneous removal of multiple trace metals [127]. A magnetic PGMA material containing dithiocarbamate and polyamine groups exhibited reported adsorption capacities of 229.41 mg g^−1^ for Pb(II) and 210.65 mg g^−1^ for Cd(II) [128], while DETA-functionalized GMA-grafted cellulose achieved 284 mg g^−1^ for Ni(II) [129]. Although these results demonstrate the potential of different functionalization strategies, the reported capacities were obtained under different experimental conditions and cannot be used to establish a direct performance ranking. Differences in solution pH, initial concentration, contact time, temperature, and material architecture must be considered when interpreting these values. Furthermore, the absence of authentic wastewater testing, residual-concentration measurements, and direct comparisons with commercial ion-exchange resins in several studies limits the assessment of their practical treatment efficiency.

Anion and oxyanion removal represents another important application of epoxy- and GMA-based materials. Aminated and quaternized GMA systems have been investigated for the capture of chromate, arsenate, phosphate, nitrate, fluoride, vanadate, molybdate, tungstate, iodide, and borate [49,53,54,55,56,83,95]. Their effectiveness is strongly influenced by solution pH, target-ion speciation, and competition from common background ions, including chloride, sulfate, carbonate, phosphate, and silicate, which may occur at substantially higher concentrations than the target contaminants. Thiourea-modified TD301 exhibited pronounced Mo(VI)/W(VI) discrimination, with a reported selectivity coefficient of 4.57 × 10^4^ at pH 1.5 [119]. As a comparative example of ion-imprinting selectivity, an As(V)-imprinted diatomite-based adsorbent exhibited selectivity coefficients of 12.14 over Cr(VI) and 27.79 over Mo(VI) [123]. Within GMA-based systems, amino-functionalized PGMA was evaluated in fixed-bed operation for vanadium removal, achieving a reported column capacity of 75.5 mg g^−1^ [130]. A DETA-functionalized magnetic GMA material combined Cr(VI) adsorption with partial reduction to Cr(III), demonstrating that contaminant removal may involve both binding and chemical transformation [133]. However, such redox-coupled processes complicate the interpretation of adsorption capacity, chromium speciation, and regeneration efficiency. In addition, many oxyanion adsorption studies lack comprehensive speciation analysis or competitive-ion experiments under representative water conditions. Consequently, high removal efficiencies measured in simplified laboratory solutions should not be interpreted as evidence of equivalent performance in natural or industrial wastewater.

Selective recovery of valuable elements represents a further application of epoxy-based ion-exchange materials, particularly for rare-earth elements, precious metals, boron, indium, uranium, and thorium [68,74,81,88,96,109,112,113,116]. Unlike contaminant removal, resource recovery requires not only effective capture but also selective separation and subsequent recovery of the target species in a concentrated and usable form. Polyaminophosphonate-functionalized PGMA demonstrated moderate discrimination between La(III) and Y(III) [112]. For comparison, a non-epoxy chitosan-based ion-imprinted hydrogel exhibited enhanced La(III) recognition through template-defined binding sites, illustrating a complementary selectivity strategy outside the principal material scope of this review [121]. Aminated PGMA microspheres have also been investigated for Pt(IV) and Pd(II) capture, while DEAE-functionalized PGMA exhibited Pd/competing-metal separation factors exceeding 30 under selected experimental conditions [101,124]. EDA-functionalized poly(GMA-co-EGDMA) enabled rapid Au(III) uptake, although partial reduction to metallic gold indicates that adsorption and chemical transformation may occur simultaneously [125]. Magnetic PEI-functionalized microspheres achieved a reported Ce(III) adsorption capacity of 179.53 mg g^−1^ [126]. These studies highlight the potential of epoxy- and GMA-based platforms for selective capture and concentration of valuable elements. Nevertheless, adsorption capacity and selectivity alone do not establish successful resource recovery. A comprehensive assessment must also demonstrate target-element recovery or product isolation, concentration efficiency, recovered-product purity, and preservation of material performance during repeated adsorption–desorption cycles. The limited reporting of these outcomes remains an important obstacle to evaluating the practical feasibility of resource recovery using epoxy-based ion-exchange materials.

## 7. Recovery Performance, Regeneration, Sustainability, and Scale-Up

Regeneration determines whether a high-capacity epoxy-based adsorbents can function as reusable ion exchangers rather than single-use materials. Acidic eluents are commonly used to protonate amine, carboxylate, phosphonate, and iminodiacetate groups, promoting the release of bound metal ions. Salt or alkaline solutions may be more suitable for anion-exchange systems, depending on the functional groups and target-ion speciation. Chelating agents such as EDTA can enhance desorption but may generate secondary metal–chelator waste streams. Several GMA-based chelating resins have demonstrated capacity retention over repeated adsorption–desorption cycles [59,60,82,85]. Magnetic GMA-based resins have been investigated for Hg(II) adsorption, wastewater treatment, and regeneration [113], while radiation-grafted sorbents have been evaluated under repeated batch and continuous-flow conditions [110]. However, regeneration performance is frequently assessed using removal efficiency or capacity retention over only a few cycles. Such measurements do not establish complete contaminant recovery or long-term material stability. A more comprehensive evaluation should include desorption efficiency, target-ion mass balance, regenerant consumption, recovery purity, polymer mass loss, ligand leaching, and changes in pore structure and mechanical integrity. These parameters are essential for distinguishing reversible adsorption from progressive material degradation (Table 4).

The sustainability of epoxy-based ion-exchange materials depends on both their synthesis and operational lifetime. Aqueous functionalization, radiation grafting, photografting, and flow-through processing offer opportunities to reduce solvent consumption or processing time, although their environmental benefits depend on the specific operating conditions [50,53,61,80,110]. Magnetic composites facilitate solid–liquid separation but often require additional inorganic precursors, surface modification, and coating steps [51,85,113,126,128]. Hydrogels and cryogels provide hydrated networks that facilitate ion transport, although their preparation and mechanical stabilization may involve substantial water and crosslinker consumption [38,39,40]. Membrane adsorbers can offer short transport pathways and potentially lower hydraulic resistance, but their practical advantages depend on flux, fouling, cleaning requirements, and service lifetime [30,50]. Therefore, high contaminant removal efficiency alone does not establish environmental sustainability. Meaningful comparisons require information on reagent toxicity, solvent and water consumption, energy demand, synthesis yield, regeneration frequency, secondary waste generation, and end-of-life management. The inconsistent reporting of these parameters currently limits quantitative life-cycle assessment and comparisons among material classes.

Scale-up requires moving beyond equilibrium adsorption capacity toward performance under realistic water-treatment conditions. In packed-bed systems, breakthrough capacity, treated bed volumes, residence time, pressure drop, and mass-transfer-zone characteristics determine operational performance. Membrane and monolithic adsorbers additionally require evaluation of permeate flux, dynamic binding capacity, hydraulic resistance, and fouling behavior. Flow-through supermacroporous composites have demonstrated the potential of convective transport for trace-metal capture [37], while membrane adsorbers can reduce transport distances, although dense grafted layers may introduce diffusion limitations [50]. Poly-GMA–glucamine systems have been evaluated in fixed-bed columns for boron recovery from reverse-osmosis streams [81,109], and amino-functionalized polymeric adsorbents have been investigated through fixed-bed modeling for vanadium removal [130]. Nevertheless, most reported materials remain at laboratory batch scale, with limited validation using authentic wastewater or direct comparisons with commercial ion-exchange resins. Future studies should integrate competitive-ion testing, extended continuous-flow operation, repeated regeneration, and recovery of concentrated target species. Process evaluation should also consider material productivity, chemical and energy consumption, operational stability, recovered-product value, and techno-economic feasibility. These interconnected requirements define the transition from laboratory adsorption measurements to practical water treatment and resource recovery (Figure 5).

Machine learning (ML) offers a potential approach to accelerating the design and optimization of epoxy- and GMA-based ion-exchange materials by identifying relationships between polymer composition, functional-group density, pore structure, and adsorption performance. Data-driven models could help predict adsorption capacity, selectivity, and regeneration behavior under different operating conditions, including variations in pH, temperature, contaminant concentration, and competing-ion composition. However, their application to epoxy/GMA-based ion exchangers remains insufficiently established, particularly because published studies differ substantially in material characterization, experimental protocols, and reporting standards. Models trained predominantly on batch adsorption data may also perform poorly under realistic wastewater conditions or continuous-flow operation. Future progress will therefore require standardized datasets that distinguish theoretical and accessible binding-site densities, incorporate competitive adsorption and regeneration measurements, and include independent experimental validation. Machine learning should be regarded as a complementary tool for identifying promising material designs rather than a substitute for mechanistic characterization and practical performance testing.

## 8. Conclusions and Future Perspectives

Epoxy- and GMA-based ion-exchange materials offer versatile platforms for water treatment and selective resource recovery. Their reactive epoxide groups enable the incorporation of diverse ion-exchange and chelating functionalities into beads, membranes, fibres, monoliths, hydrogels, and composites. These materials have been investigated for the removal of metal ions, inorganic oxyanions, nutrients, and organic contaminants, as well as the recovery of valuable elements. However, high functional-group density does not necessarily translate into high adsorption capacity. Site accessibility, hydration, pore connectivity, crosslinking, and mechanical stability collectively determine the fraction of binding sites that can contribute to effective ion uptake. Consequently, theoretical ligand density and model-derived maximum capacity should not be treated as reliable indicators of practical treatment performance.

A central limitation of the existing literature is the insufficient experimental verification of binding mechanisms and structure–performance relationships. Functionalization is frequently confirmed through FTIR without quantitative assessment of accessible ion-exchange capacity, ligand distribution, or post-adsorption chemical states. Similarly, ion exchange, coordination, and chemisorption are often inferred from equilibrium and kinetic model fitting rather than counter-ion balances or direct evidence of metal–ligand interactions. Quantitative mass-transfer parameters also remain inadequately reported. Furthermore, most adsorption studies rely on single-solute laboratory solutions, with limited investigation of competitive ions, natural organic matter, authentic water matrices, and continuous-flow conditions. These shortcomings restrict meaningful comparisons among materials and complicate the translation of laboratory capacities into operational performance.

Future research should prioritize quantitative verification of functional-group accessibility, experimentally supported binding mechanisms, and standardized adsorption measurements under realistic treatment conditions. Competitive-ion experiments, fixed-bed breakthrough studies, and direct benchmarking against commercial ion-exchange resins are particularly important. Regeneration assessments should extend beyond short-term capacity retention to include desorption efficiency, recovered-product purity, chemical consumption, ligand leaching, and long-term material stability. Sustainable development also requires safer functionalization routes, reduced solvent and energy consumption, and systematic life-cycle and techno-economic evaluation. Ultimately, progress will depend on integrating molecular design, accessible capacity, mass transfer, regeneration, and process engineering into a unified framework. Such an approach is essential for translating epoxy-based ion-exchange materials from laboratory sorbents into reliable technologies for water purification and resource recovery.

## Figures and Tables

**Table 1:** Characterization methods and representative findings for structure–property verification of epoxy- and GMA-based ion-exchange materials.

Characterization Category	Analytical Technique	Material/System	Property or Feature Verified	Key Reported Value or Finding	Ref.
Spectroscopic confirmation of epoxide conversion					
Epoxide conversion	FTIR	Radiation-polymerized PGMA microspheres converted into an anion-exchange resin for I^−^ uptake	Consumption of pendant epoxide groups and introduction of anion-exchange sites	Disappearance of characteristic epoxy-ring absorption bands; no numerical conversion percentage reported in the accessible record	[95]
Epoxide conversion and sulfonation	FTIR	Sulfonated PGMA–DVB microspheres	Epoxide consumption and introduction of sulfonate groups	FTIR confirmed sulfonation; maximum Cu^2+^ adsorption capacity of 75.08 mg g^−1^ was reported separately	[34]
Epoxide conversion	FTIR and epoxy-content determination	GMA-co-nBA microspheres modified with amines for enzyme immobilization	Consumption of epoxide groups during amination	Reduced epoxy-ring FTIR intensity supported ring opening; no numerical conversion percentage reported in the accessible abstract	[114]
Epoxide conversion	FTIR	Post-modified DMN-co-GMA copolymer microspheres	Chemical changes following epoxide-ring opening	FTIR changes and a decrease in BET surface area supported successful modification	[115]
Sulfonation and epoxide conversion	FTIR and Raman spectroscopy	Sulfonated poly(acrylonitrile-co-GMA)/PVC proton-exchange membranes	Introduction of –SO_3_H groups and preservation of the composite structure	FTIR and Raman spectra supported sulfonation; IEC values of 0.18–0.48 meq g^−1^ were reported (row 16)	[97]
Epoxide conversion and amination	FTIR-ATR and solid-state ^13^C NMR	DETA-functionalized GMA–EGDMA porous monolith	Epoxide-ring opening and introduction of –NH_2_ and –NH– groups	Complementary FTIR-ATR and solid-state NMR measurements supported amine functionalization	[75]
Epoxide conversion and elemental incorporation	FTIR and elemental analysis	GMA-grafted nonwoven cotton functionalized with EDA	Ring opening and introduction of surface amine groups	FTIR, SEM, EDX, XPS, and elemental analysis collectively supported EDA functionalization	[98]
Multistep functionalization	FTIR	PGMA modified with iminodiacetate and imino-phosphonate groups	Epoxide consumption and introduction of carboxylate and phosphonate ligands	FTIR supported multistep functionalization; comparative Nd^3+^ capacities are presented in row 18	[96]
Surface and bulk elemental or chemical-state analysis					
Surface chemistry and bulk metal uptake	XPS and ICP-OES	Functionalized macroporous poly(GMA-co-EDMA) sorbent	Surface chemical states and metal–ligand coordination	XPS supported surface functionalization and coordination of Cu^2+^, Cd^2+^, and Pb^2+^; ICP-OES quantified bulk metal uptake	[100]
Ligand loading and coordination	XPS and CHN elemental analysis	Amino-modified monodisperse PGMA microspheres for Pt(IV) and Pd(II) uptake	Amine incorporation and N–metal coordination	Elemental analysis quantified amine loading, while XPS supported N–Pd coordination	[101]
Surface and bulk composition	XPS and CHN elemental analysis	Functionalized PGMA for U(VI) uptake	Participation of nitrogen-containing groups in U(VI) binding	XPS supported coordination through nitrogen-containing groups; CHN analysis verified bulk functional-group incorporation	[102]
Complementary surface and bulk verification	XPS, SEM, EDX, FTIR, and elemental analysis	EDA-modified GMA-grafted nonwoven cotton	GMA grafting, nitrogen incorporation, and surface modification	Agreement among surface-sensitive and bulk measurements provided stronger evidence of amine functionalization than any single technique	[98]
Epoxide content and ion-exchange capacity determination					
Residual epoxide content	Titrimetric epoxide determination	Poly(GMA-co-EGDMA) copolymers prepared under different reaction conditions	Absolute epoxide content as a function of monomer, crosslinker, and porogen composition	Titration quantified residual epoxide groups and enabled comparison with porosity and thermal-residue measurements	[84]
Intrinsic ion-exchange capacity	IEC measurement	Crosslinked anion-exchange membranes prepared from diglycidyl ether epoxy resin and quaternized PEI	Total anion-exchange capacity and its relationship with hydration and transport	IEC ranged from 1.5 to 3.7 mmol g^−1^ in the Cl^−^ form at 20 °C; water uptake ranged from 20.0% to 330%	[30]
Intrinsic ion-exchange capacity	IEC measurement with FTIR and Raman verification	Sulfonated poly(acrylonitrile-co-GMA)/PVC membranes	IEC produced by sulfonation	IEC values ranged from 0.18 to 0.48 meq g^−1^; methanol permeability was reported as 8.7 × 10^−7^ cm^2^ s^−1^	[97]
Pollutant-specific accessible capacity and regeneration	Batch adsorption and desorption with 0.5 M HNO_3_	Multifunctionalized poly(GMA) for Y^3+^ and La^3+^ recovery	Rare-earth uptake and acid regenerability	Desorption exceeded 93% for La^3+^ and 95% for Y^3+^ during six adsorption–desorption cycles	[112]
Ligand-dependent accessible capacity	Batch Nd^3+^ adsorption	PGMA modified with iminodiacetate and imino-phosphonate functionalities	Comparative accessibility and effectiveness of chelating groups	Reported capacities were 0.645 mmol g^−1^ for IDA-PGMA, 0.736 mmol g^−1^ for amino-PGMA, and 0.304 mmol g^−1^ for N-PGMA; HNO_3_ elution exceeded 91%	[96]
Pollutant-specific accessible capacity	Batch Cu^2+^ adsorption	Sulfonated PGMA–DVB microspheres	Accessible sulfonate functionality under the selected test conditions	Maximum Langmuir capacity was 75.08 mg Cu^2+^ g^−1^	[34]
Pollutant-specific accessible capacity	Batch Th^4+^ adsorption	Amine-functionalized GMA resins RI and RII	Actinide-binding capacity and temperature dependence	Maximum capacities of 50 and 64 mg Th^4+^ g^−1^ were reported for RI and RII, respectively	[116]
Morphology, pore structure, and surface area					
Dry-state surface area and pore-size distribution	N_2_ adsorption and BET analysis	Crosslinked DVB–GMA porous polymers containing DVB/GMA ratios of 75:25 and 50:50	Influence of composition on mesoporosity and surface area	The 75:25 materials exhibited BET areas of approximately 320–370 m^2^ g^−1^, whereas the 50:50 materials showed approximately 140–190 m^2^ g^−1^; pore modes occurred near 3.7 and 5.5–7.5 nm	[72]
Dry-state surface area	N_2_ adsorption and BET analysis	Histidine-modified poly(GMA-co-EGDMA) sorbent	Retention of porous structure after functionalization	A BET surface area of approximately 90 m^2^ g^−1^ was reported	[103]
Particle morphology and size	SEM with particle-size analysis	Sulfonated PGMA–DVB microspheres	Particle uniformity and morphological changes following DVB incorporation and sulfonation	Mean particle diameters were 2.18 µm for PGMA and 2.52 µm for PGMA–DVB; DVB increased surface roughness	[34]
Surface morphology and wettability	SEM, AFM, CLSM, and contact-angle measurements	Multilayer dextran-decorated poly(GMA-co-DVB) matrices	Surface modification and hydrophilicity	Dextran decoration reduced the reported contact angle from 153° to approximately 0°	[105]
Crosslinking-dependent porosity	Mercury intrusion porosimetry and SEM	Macroporous amino-functionalized GMA-based copolymer beads	Effects of crosslinker concentration and functionalization on pore structure	Greater crosslinker content increased surface area and reduced pore diameter; SEM supported spherical particle morphology	[104]
Dry porosity versus wet operational behavior	BET/t-plot analysis and wet-bed dynamic-capacity measurements	Polar-modified post-crosslinked resin prepared using different porogens	Relationship between dry-state porosity and dynamic wet-state performance	Dynamic saturation capacity reached 55.4 mg mL^−1^ of wet resin, demonstrating the need to combine dry and hydrated-state measurements	[77]
Drying-induced porosity artifacts	BET analysis with thermal- and freeze-drying comparison	Macroreticular anion exchanger containing Cu_2_O or CuO deposits	Influence of sample preparation on measured porosity	Thermal drying caused particle shrinkage and pore collapse, whereas freeze-drying better preserved the porous structure	[106]
Hydrated behavior, wettability, and thermal stability					
Water uptake and thermal behavior	Gravimetric water uptake and TGA	Crosslinked epoxy-resin anion-exchange membranes	Hydration capacity and thermal stability	Water uptake ranged from 20.0% to 330% across the membrane series; TGA and mechanical testing complemented the hydration measurements	[30]
Thermal stability	TGA and DSC	Organic–inorganic hybrid membrane based on an epoxy-silane component	Resistance of the hybridized material to thermal degradation	Heat resistance above 300 °C was reported; detailed onset and mass-loss values were unavailable in the accessible record	[43]
Thermal stability and hydration	TGA and gravimetric water uptake	Sulfonated PGMA-grafted Nafion membranes	Effects of grafting and sulfonation on thermal behavior and hydration	Greater water uptake and thermal stability than unmodified Nafion were reported qualitatively	[108]
Thermal stability	TGA	Amine-functionalized GMA resins for Th^4+^ extraction	Thermal behavior of the functionalized resin	Thermal stability was evaluated, but numerical onset temperatures were unavailable in the accessible record	[116]
Dynamic performance, regeneration, and operational stability					
Dynamic capacity, scale-up, and cycling	Fixed-bed breakthrough, BDST modelling, and repeated-cycle testing	Poly-GMA–glucamine column for boron recovery from seawater reverse-osmosis concentrate	Dynamic bed capacity, critical bed depth, enrichment, and regenerability	BDST capacities of 3872–3999 mg L^−1^ and critical bed depths of 4.7–9.2 mm were reported. Regeneration with 1.0 M HCl produced 97.5% desorption, 78-fold enrichment, and greater than 95% capacity retention over five cycles	[109]
Dynamic breakthrough performance	Fixed-bed column experiments and breakthrough modelling	Radiation-grafted amino-type PP-g-GMA material for Cr(VI) and Cd(II) removal	Treated bed volumes and flow-dependent breakthrough	More than 4000 bed volumes of a 3 mg L^−1^ Cr(VI) solution were reportedly treated before breakthrough at 155 BV h^−1^	[110]
Regeneration stability	Fixed-bed adsorption–desorption cycling	Radiation-grafted PP-g-GMA adsorbent	Regenerant effectiveness and capacity retention	Cr(VI) was desorbed using 2 M NaOH, whereas Cd(II) was stripped with organic acids; adsorption efficiency remained above 94% over five cycles	[110]
Dynamic removal and regeneration	Fixed-bed column and NaCl regeneration	Aminated poly-GMA beads for phosphorus removal	Dynamic phosphate uptake, competing-anion effects, and salt regeneration	NaCl washing reportedly did not substantially reduce capacity, whereas sulfate was identified as an important competing ion	[111]
Batch regeneration	Four adsorption–desorption cycles	Magnetic GMA-based resin containing a mercury-binding ligand	Reusability and applicability to industrial wastewater	The sorbent remained effective through at least four cycles; the regenerant was not specified in the accessible record	[113]
Rare-earth desorption and recycling	Batch adsorption and HNO_3_ desorption	Multifunctionalized poly(GMA) resin for Y^3+^ and La^3+^ recovery	Acid-regeneration efficiency over repeated cycles	Desorption remained above 93% for La^3+^ and 95% for Y^3+^ during six cycles using 0.5 M HNO_3_	[112]
Low-pressure dynamic binding	UV grafting and fixed-bed dynamic-capacity testing	GMA-grafted PES membrane adsorber for Cu^2+^ capture	Degree of grafting, dynamic capacity, flux, and operating pressure	Degrees of grafting of 9%, 19%, and 41% were examined; dynamic capacity reached 64.05 ± 0.60 mg Cu^2+^ g^−1^ of grafted material at 135 L m^−2^ h^−1^ and below 0.15 bar	[50]

AFM, atomic force microscopy; ATR, attenuated total reflectance; BDST, bed-depth service-time; BET, Brunauer–Emmett–Teller; BSA, bovine serum albumin; BV, bed volume; CHN, carbon–hydrogen–nitrogen elemental analysis; CLSM, confocal laser scanning microscopy; DETA, diethylenetriamine; DMN, dimethacrylate-based network; DVB, divinylbenzene; EDA, ethylenediamine; EDMA/EGDMA, ethylene glycol dimethacrylate; EDX, energy-dispersive X-ray spectroscopy; FTIR, Fourier-transform infrared spectroscopy; GMA, glycidyl methacrylate; ICP-OES, inductively coupled plasma optical emission spectroscopy; IDA, iminodiacetate; IEC, ion-exchange capacity; NMR, nuclear magnetic resonance; PEI, polyethylenimine; PES, polyethersulfone; PGMA, poly(glycidyl methacrylate); PP, polypropylene; PVC, poly(vinyl chloride); SEM, scanning electron microscopy; TGA, thermogravimetric analysis; XPS, X-ray photoelectron spectroscopy.

## Data Availability

No new data were created or analyzed in this study. Data sharing is not applicable to this article.

## Supplementary Materials

The following supporting information can be downloaded at: https://www.mdpi.com/article/10.3390/polym18212710/s1, Table S1: Database Search Strategies, Search Parameters, Retrieval Counts, and Search Dates; Table S2: Title and Abstract Screening Results and Relevance Assessment of Retrieved Studies on Epoxy-Based Ion-Exchange Materials.

## Abbreviations

The following abbreviations are used in this manuscript:

ACN	Acrylonitrile
AFM	Atomic force microscopy
AGET-ATRP	Activators generated by electron transfer–atom transfer radical polymerization
APTMS	(3-Aminopropyl)trimethoxysilane
ATR	Attenuated total reflectance
ATRP	Atom transfer radical polymerization
BADGE	Bisphenol A diglycidyl ether
BDST	Bed-depth service-time
BET	Brunauer–Emmett–Teller
BSA	Bovine serum albumin
BV	Bed volume
CEC	Cation-exchange capacity
CHN	Carbon, hydrogen, and nitrogen elemental analysis
CLSM	Confocal laser scanning microscopy
COS	Chitosan oligosaccharide
CSSNa	Sodium dithiocarbamate-functionalized component (see note below)
DEAE	Diethylaminoethyl
DETA	Diethylenetriamine
DFT	Density functional theory
DGEBA	Diglycidyl ether of bisphenol A
DMN	Dimethacrylate-based network
DSC	Differential scanning calorimetry
DTPA	Diethylenetriaminepentaacetic acid
DVB	Divinylbenzene
EDA	Ethylenediamine
EDMA	Ethylene glycol dimethacrylate
EDS/EDX	Energy-dispersive X-ray spectroscopy
EGDMA	Ethylene glycol dimethacrylate
EXAFS	Extended X-ray absorption fine structure
FTIR	Fourier-transform infrared spectroscopy
GMA	Glycidyl methacrylate
HSAB	Hard and soft acids and bases
ICP-OES	Inductively coupled plasma optical emission spectroscopy
IDA	Iminodiacetate
IEC	Ion-exchange capacity
IPD	Intraparticle diffusion
IUPAC	International Union of Pure and Applied Chemistry
LFD	Liquid-film diffusion
MB	Methylene blue
MBA	N,N′-Methylenebisacrylamide
MTHPA	Methyltetrahydrophthalic anhydride
NMR	Nuclear magnetic resonance
NR	Not reported
PEI	Polyethylenimine
PES	Polyethersulfone
PET	Poly(ethylene terephthalate)
PFO	Pseudo-first-order
PGMA	Poly(glycidyl methacrylate)
PMMA	Poly(methyl methacrylate)
PP	Polypropylene
PPG	Polypropylene glycol
PS-DVB	Polystyrene–divinylbenzene
PSO	Pseudo-second-order
PVC	Poly(vinyl chloride)
PVDF	Poly(vinylidene fluoride)
SEC	Static exchange capacity
SEM	Scanning electron microscopy
SMZ	Sulfamethoxazole
SPGMA	Sulfonated poly(glycidyl methacrylate)
TETA	Triethylenetetramine
TGA	Thermogravimetric analysis
UV	Ultraviolet
XPS	X-ray photoelectron spectroscopy
XRD	X-ray diffraction
